# In Vitro Antimicrobial and Antiproliferative Activities of the Root Bark Extract and Isolated Chemical Constituents of *Zanthoxylum paracanthum* Kokwaro (Rutaceae)

**DOI:** 10.3390/plants9070920

**Published:** 2020-07-21

**Authors:** Magrate M. Kaigongi, Catherine W. Lukhoba, Souaibou Yaouba, Nokwanda P. Makunga, Joseph Githiomi, Abiy Yenesew

**Affiliations:** 1 School of Biological Sciences, University of Nairobi, P.O. Box 30197, Nairobi 00100, Kenya; clukhoba@yahoo.co.uk; 2 Kenya Forestry Research Institute, P.O. Box 20412, Nairobi 00200, Kenya; josephgithiomi@ymail.com; 3 Department of Chemistry, University of Nairobi, P.O. Box 30197, Nairobi 00100, Kenya; souaibouyaouba@yahoo.fr (S.Y.); ayenesew@uonbi.ac.ke (A.Y.); 4 Department of Botany and Zoology, Stellenbosch University, Private Bag X1, Matieland 7602, South Africa

**Keywords:** alkaloids, antimicrobial activity, anticancer activity, canthinones, medicinal plant, phytochemistry, traditional medicine

## Abstract

*Zanthoxylum paracanthum* Kokwaro (Rutaceae) is an endemic Kenyan and Tanzanian plant used in folk medicine by local populations. Although other *Zanthoxylum* species have been studied, only *Z. paracantum* stem extracts have been profiled, even though the roots are also used as herbal remedies. As root extracts may be another source of pharmaceutical compounds, the CH_2_Cl_2_/MeOH (1:1) root bark extract was studied in this report. Eight root bark compounds were isolated and their structural identities were confirmed by mass spectrometry (MS) and nuclear magnetic resonance (NMR) (using COSY, HSQC, NOESY and HMBC) analyses. The structural identities were determined as follows: the fatty acid—myristic acid (**1**); the sterol—stigmasterol (**2**); the lignan—sesamin (**3**); two β-carboline alkaloids—10-methoxycanthin-6-one (**6**) and canthin-6-one (**7**); and three phenanthridine alkaloids—8-acetonyldihydrochelerythrine (**4**), arnottianamide (**5**) and 8-oxochelerythrine (**8**). Some of these compounds were identified in the species for the first time. These compounds and the extract were then tested in vitro against methicillin-resistant *Staphylococcus aureus* (MRSA), *Escherichia coli* (ATCC 25922), *Staphylococcus aureus* (ATCC 29213) and *Candida albicans* (ATCC 10231) before tests for antiproliferative activity against the human breast cancer (HCC 1395), human prostate cancer (DU 145) and normal (Vero E6) cell lines were conducted. Minimum inhibition concentration values of 3.91, 1.95, 0.98 and 7.81 µg/mL against MRSA, *S. aureus*, *E. coli* and *C. albicans*, respectively, were recorded. Among the isolates, canthin-6-one was the most active, followed by 10-methoxycanthin-6-one. The root extract and some of the compounds also had antiproliferative activity against the HCC 1395 cell line. Stigmasterol and canthin-6-one had IC_50_ values of 7.2 and 0.42. The root bark extract also showed activity, at 8.12 µg/mL, against the HCC 1395 cells. Out of the chemical isolates, 10-methoxycanthin-6-one and canthin-6-one showed the strongest inhibition of the DU 145 cells. The root extract had significant antimicrobial and antiproliferative activities, supporting the traditional use of this plant in treating microbial infections and cancer-related ailments.

## 1. Introduction

Medicinal plants remain an important source of pharmaceutics, and in communities where access to healthcare is limited, plant-based medications have a high proportion of use [1]. In Africa, including Kenya, the use of herbal medicines to treat different ailments, including infectious diseases and cancers, is common [2]. Microbial diseases are often implicated in the onset of cancers, and compounds that may have both antimicrobial and anticancer effects are thus sought after. In Kenya, breast, cervical, oesophageal, colon, stomach and ovarian cancers occur at higher incidents in women, while in men, prostate, oesophageal, colon, stomach, oral and liver cancers are most prevalent [3]. With urbanisation, higher levels of cancers are becoming more apparent, and a recent study in 2019 showed that there are more female cancer cases than male; 60% and 56.4%, respectively [4]. There is thus a global commitment to finding new drug entities that are effective in reducing the proliferation of cancers [5]. In the search for new plant-derived pharmaceuticals, the candidate drug should demonstrate selectivity by having low levels of toxicity to normal cell lines, and superior cytotoxicity towards cancer cell lines [6]. In general, natural products with pharmacological effects can become lead structures in the preparation of semi-synthetic and synthetic drugs [7]. In this regard, the plants used in traditional medicine remain sources of such structures [8]. Incidentally, a large fraction of medicinal plants lack the scientific backup to substantiate their traditional uses [1]. Additionally, their toxicities and safe dosages are unknown [9]. *Zanthoxylum paracanthum* Kokwaro (Family Rutaceae) is a shrub or a small tree occurring in the coastal areas of Kenya and Tanzania, and it has a long history as a traditional medicine in the areas where it is found [10]. Although some of the other *Zanthoxylum* species are quite well studied, little ethnobotanical information exists because of their narrow distribution. Communities living in the coastal areas of Kenya use stem and root extracts of this plant in the management of tumors and other related diseases [11], but thus far, only stem extracts have been chemically profiled. In Tanzania, the leaves are used as an indigenous vegetable, as they have a high nutrient content and it is claimed they cure gastrointestinal problems such as diarrhoea [12], suggesting the antimicrobial potential of the plant. The isolation of three new alkaloids, zanthoxoaporphines A–C with larvicidal activity against the third-instar *Anopheles gambiae* larvae [13], from a *Z. paracanthum* stem extract has shown the possibility of discovering new compounds from this species. In another study, canthin-6-one, isolated from the stem bark extract of *Z. paracanthum*, was found to be active against both drug-sensitive and multidrug-resistant leukemia cell lines [11].

As there are close to 250 *Zanthoxylum* species, only some plants from this taxon have been investigated for their chemical and pharmacological properties. The antimicrobial and cytotoxicity effects have been studied in species such as *Z. budruga* [14], *Z. chalybeum* [15], *Z. leprieurii* and *Z. zanthoxyloides* [16]. As the root bark extract of Z. *paracanthum* has never been studied in this way before, we identified this extract as a possible new source of bioactive compounds. Eight compounds were purified from the root bark extract, and here, the in vitro antimicrobial and antiproliferative activities of the root bark extract and the eight chemical isolates of *Z. paracanthum* are reported. 

## 2. Results and Discussion

### 2.1. Identification of the Isolated Compounds

**Figure 1:**
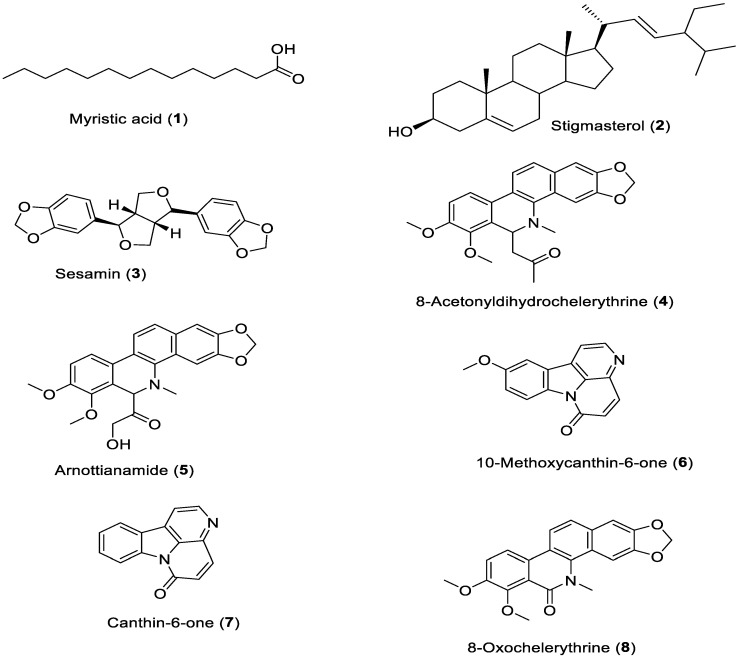
Structures of compounds (**1–8**) isolated from root bark of *Zanthoxylum paracanthum.*

Eight known compounds were isolated (Figure 1) and identified, by MS and NMR (using COSY, HSQC, NOESY and HMBC) analyses (see Supplementary Materials) as myristic acid (**1**) [17], stigmasterol (**2**) [18], sesamin (**3**) [11], 8-acetonyldihydrochelerythrine (**4**) [19], arnottianamide (**5**) [20], 10-methoxycanthin-6-one (**6**) [21], canthin-6-one (**7**) [11] and 8-oxochelerythrine (**8**) [22].

### 2.2. Antimicrobial Activity

The CH_2_Cl_2_/CH_3_OH (1:1) extract from the root bark of *Z. paracanthum* had high antimicrobial activity against all the tested microbes (Table 1). The minimal inhibition concentration (MIC) values of this extract were 3.91, 1.95, 0.98 and 7.81 µg/mL, against MRSA, *E. coli*, *S. aureus* and *C. albicans*, respectively. This is the first report on the antimicrobial activity of *Z. paracanthum*. The activity level observed here is similar to the antimicrobial activities reported for other Kenyan *Zanthoxylum* species. *Z. chalybeum* elicited a broad-spectrum antimicrobial activity against *Bacillus cereus*, *Pseudomonas aeruginosa*, MRSA and *Candida albicans* [23]. The high and broad-spectrum antimicrobial activities of *Z. gilletii* and *Z. holstzianum* have been reported [24,25]. These studies support the traditional use of *Zanthoxylum* species in Kenya in the broad-spectrum treatment of microbial infections, and because the extract exhibited good and promising antimicrobial effects, the compounds that exhibit such activity were of interest. 

The eight isolated phytochemicals were tested for antimicrobial activity, of which myristic acid (**1**) had no activity against all the tested microbes with MIC values of more than 1000 µg/mL (Table 1). This is in line with the previous antimicrobial studies on this saturated fatty acid, myristic acid, which recorded no activity against MRSA [26]. Plant sterols are often a good source of antimicrobial agents, and in this study stigmasterol (**2**) obtained from the root bark extract did not inhibit the growth of MRSA and *C. albicans*, but showed moderate activity against the growth of *S. aureus* and *E. coli*, with MIC values of 62.50 µg/mL and 15.63 µg/mL, respectively. Elsewhere, a study on the antimicrobial activity of stigmasterol indicated its activity mainly against *Streptococcus mutans* and *S. sobrinus* strains, but with very limited activity [27]. Sesamin (**3**) is a lignan, and occurs in a wide variety of plant species, including *Sesamum indicum* L. [28]. Sesamin had no activity against MRSA, *E. coli* or *C. albicans*, but had an inhibitory effect against the growth of *S. aureus*, with an MIC value of 500 µg/mL. Previous studies on the antimicrobial and antioxidant properties of sesamin revealed low antibacterial activity [29]. Additionally, sesamin did not inhibit the growth of MRSA, the resistant strain of *S. aureus* [30].

One of the most potent compounds isolated in this study is 8-acetonyldihydrochelerythrine (**4**), which inhibited the growth of all four microbes, with MIC values of 31.25 µg/mL against MRSA, 15.63 µg/mL against *S. aureus* and *E. coli*, and 62.50 µg/mL against *C. albicans.* These results are in agreement with what has been reported for the same compound isolated from *Zanthoxylum rhetsa* (Roxb.) DC. roots, which exhibited strong activity against MRSA (MIC = 8 μg/mL) and moderate activity against *E. coli* (MIC = 16 μg/mL) [31]. Arnottianamide (**5**) was among the isolated compounds that did not inhibit the growth of all the microbes tested (MIC above 1000 µg/mL), which is in agreement with the reports that indicated that this compound did not exhibit any antimicrobial activity against the microbes tested [32,33].

*Zanthoxylum* species are regarded as good sources of alkaloids, and here we have isolated and determined the antimicrobial effects of the alkaloid 10-methoxycanthin-6-one (**6**). This compound possessed antimicrobial activity against all the microorganisms tested. The MIC values of this compound against each microbe were 3.91 µg/mL against MRSA, 1.95 µg/mL against *S. aureus*, 3.91 µg/mL against *E. coli* and 7.81 µg/mL against *C. albicans*. Previous studies illustrate that this compound exhibited high inhibitory activities against *Bacillus cereus, Bacillus subtilis, Ralstonia solanacearum* and *Pseudomonas syringae*, with MIC values between 3.91 and 31.25 μg/mL, and significant antifungal activity against *Fusarium graminearum* [34]. This is the first report on the antimicrobial activity of this compound against the four tested microbes. A study on structure–activity relationship (SAR) showed that the high antimicrobial activity is conferred by the aliphatic ester derivatives of 10-hydroxycanthin-6-one [35]. The related alkaloid, canthin-6-one (**7**), exhibited the highest antimicrobial activity (Table 1) against all of the tested microbes, with activity levels similar to those of the positive controls (*p* ≥ 0.05). The recorded MIC values for this compound were 0.97 µg/mL for MRSA, 0.48 µg/mL for *S. aureus*, 1.95 µg/mL for *E. coli* and 3.91 µg/mL against *C. albicans*. Soriano-Agatón et al. [36] reported the antifungal activity of four naturally occurring canthin-6-one derivatives against five pathogenic fungi. However, the structure–activity relationship (SAR) for the antifungal activity remained unclear. A high antimicrobial activity of canthin-6-one against *Staphylococcus aureus* and *Saccharomyces cerevisiae* has also been reported [37].

Finally, the alkaloid 8-oxochelerythrine (**8**) had significant antimicrobial activity against all the tested microbes, with MIC values of 62.5 µg/mL against MRSA, 31.25 µg/mL against *S. aureus*, 3.91 µg/mL against *E. coli* and 15.63 µg/mL against *C. albicans*. 8-Oxochelerythrine had considerable inhibitory activity against Clostridium sporogenes (MIC 0.91 μM) and Streptococcus pyogenes (MIC 3.64 μM) [38]. The antimicrobial activity of 8-oxochelerythrine is attributed to the presence of two methoxy groups at C-7 and C-8 of this compound [39]. 

### 2.3. Antiproliferative Activity

The antiproliferative activities of the samples were determined and the IC_50_ values are given in Table 2. The criterion for reporting antiproliferative activity for natural products was based on in vitro cytotoxicity, after the exposure of the cells to the samples for 72 h, as recommended by the U.S. National Cancer Institute (NCI). An IC_50_ value lower than 20 µg/mL for crude extracts, and an IC_50_ value less than 4 µg/mL for pure compounds, is considered to be highly antiproliferative [40]. The selective inhibitory activity of the samples was determined and expressed as the selectivity index (SI). The SI values demonstrate the ability of samples to kill cancerous cells without significantly affecting the normal cells. A high SI value depicts high selectivity. Natural products with SI ≥ 2 are considered to be highly selective, while SI <2 indicates less selectivity [41]. A normal cell line, Vero E6, widely used in toxicology, virology and pharmacology research and in the testing of new vaccines and diagnostic reagents in industry [42], was used in this study. In particular, these cells have been used as models to evaluate the toxicity of compounds of different natures, either chemical or microbial toxins, and are routinely used as a part of screening programs [43]. 

**Figure 2:**
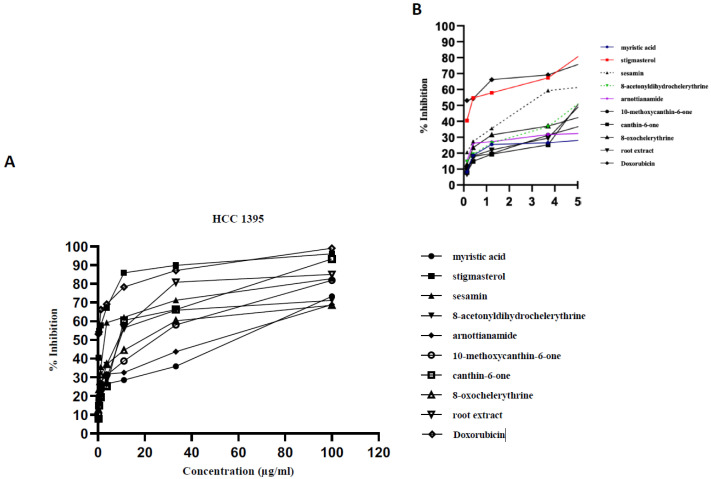
Antiproliferative activity of the tested samples against the human breast cancer cell line (HCC1395). Antiproliferative activity of the tested samples against various cancer cell lines recoded as % growth inhibition. (**A**) Biphasic growth curve of dose-dependent anti-proliferation effects: 0–100 µg/mL. (**B**) Effect of test samples at low concentrations: 0–5 µg/mL.

**Figure 3:**
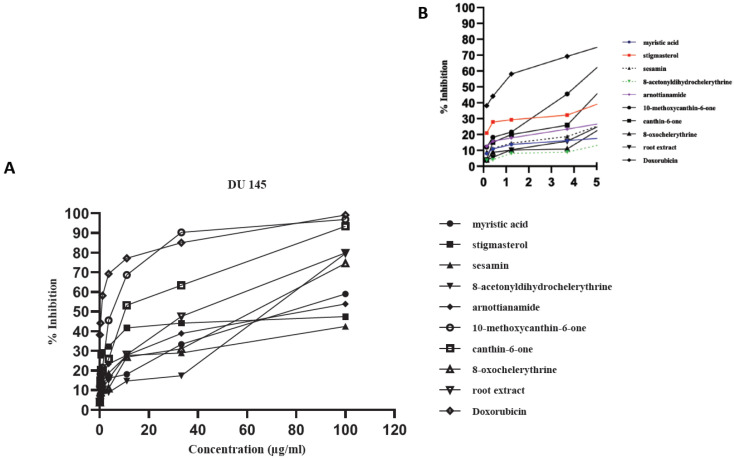
Antiproliferative activity of the tested samples against the human prostate cancer cell line (DU 145). Antiproliferative activity of the tested samples against various cancer cell lines recoded as % growth inhibition. (**A**) Biphasic growth curve of dose-dependent anti-proliferation effects: 0–100 µg/mL. (**B**) Effect of test samples at low concentrations: 0–5 µg/mL.

**Figure 4:**
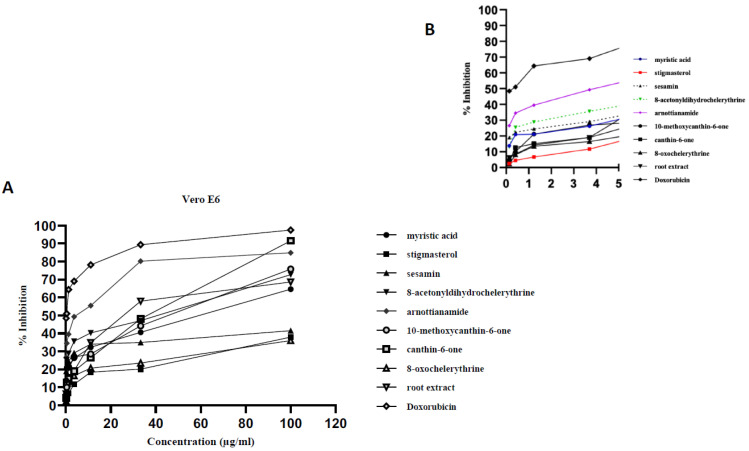
Antiproliferative activity of the tested samples against the normal cell line (Vero E6). Antiproliferative activity of the tested samples against various cancer cell lines recoded as % growth inhibition. (**A**) Biphasic growth curve of dose-dependent anti-proliferation effects: 0–100 µg/mL. (**B**) Effect of test samples at low concentrations: 0–5 µg/mL.

All the compounds exhibited concentration-dependent cytotoxicity levels (Figure 2, Figure 3 and Figure 4). The antiproliferation effects show a good correlation with the concentrations supplied to the different cell types. Some samples were highly effective against the cancer cells, with high inhibition percentages (above 70%) being recorded (Figure 2A and Figure 3A). The dose dependent curve is biphasic, with a sharp (exponential) linear relationship that is more evident as increasing concentrations of the extract or compounds are included in the growth medium. The higher concentrations of the test samples appear to be correlated with the asymptotic slope of the curve. The effect of lower concentrations, below 4 µg/mL for tested samples, is more clearly shown by Figure 2B, Figure 3B, Figure 4B. In some cases, the root bark extract and some of the compounds were highly active at these low concentrations.

The root bark extract of *Z. paracanthum* was highly antiproliferative and selective against the HCC 1395 cell line, with an IC_50_ value of 7.27 ± 0.0 µg/mL, and the SI value was recorded at 3.89 (Table 2). The extract was found to be toxic to normal cells (Vero E6), whilst it moderately inhibited the growth of DU 145 cancer cells with an IC_50_ of 53.21 ± 1.2 µg/mL, and was less selective, with an SI value of 0.51. This is the first report on the antimicrobial and anticancer activity of the root bark extract of *Z. paracanthum*. 

The pharmacological effects of the root bark extract of *Z. paracanthum* in this study could be due to the synergistic interactions of the different phytochemicals present in the extract [44] that function on the cell, leading to growth inhibition and even perhaps the apoptosis of microbial and cancer cells, respectively [45]. Studies carried out on the leaf extract of *Zanthoxylum armatum* DC indicated a high anticancer activity against the human cervical cell line [46], while in vitro bioassays of *Z. leprieurii* and *Z. zanthoxyloides* exhibited moderate anticancer and antimicrobial activities [16].

A moderate inhibition of cancer cells was observed with myristic acid (**1**) against the tested cell lines. The CC_50_ value for Vero E6 was 64.86 ± 0.51 µg/mL, while the IC_50_ values for HCC 1395 and DU 145 were 57.71 ± 1.2 µg/mL and 80.24 ± 0.12 µg/mL, respectively. The selectivity index for the human breast cancer cell line, HCC 1395, was 1.12, while that of the human prostate cancer cell line, DU 145, was 0.81. Myristic acid was therefore less selective against the tested cancer cell lines used in this study (SI values were ≤2). This was also observed previously in a study that investigated the effects of branching on fatty acid biosynthesis of human breast cancer cells, wherein myristic acid was found to be cytotoxic to the breast cancer cells [47]. 

The activity of stigmasterol (**2**) in the anticancer assays (Table 2) is not surprising. It is interestingly to note that, overall, the potency of this compound in preventing cancer growth was greatest in relation to the human breast cancer cell line (HCC1395). This compound was also effective in inhibiting the growth of the other cancer cell lines, with the human prostate cancer cell line (DU 145) showing a reduced response as compared to the breast cancer cell line, but it was less active against Vero E6. The CC_50_ value for E6 was 123.88 ± 0.00 µg/mL, while the IC_50_ values for HCC 1395 and DU 145 were 0.42 ± 0.1 µg/mL and 140.49 ± 1.27 µg/mL, respectively. The selectivity index for the human breast cancer cell line, HCC 1395, was 294.94. A such, stigmasterol was highly selective against HCC 1395, and less selective against the human prostate cancer cell line, DU 145, where the SI value was 0.88. This compound is a good candidate for further research on the control of breast cancer, as it has a very low IC_50_ value against HCC1395 as well as a very high selectivity index [48]. In a previous study, the antitumor activity of stigmasterol was evaluated against Ehrlich Ascites Carcinoma (EAC) in swiss albino mice, and the compound was able to decrease the tumor volume and improve the life span of the mice [49]. 

Lignans have become fascinating as lead compounds with anticancer activity, and there is thus increased interest in them from natural product scientists [50,51]. The lignan, sesamin (**3**), lacked significant antiproliferative activity against the normal cells Vero E6 (with a CC_50_ value of 135.31 ± 0.12 µg/mL) and DU 145 (with an IC_50_ of 115.06 ± 0.03 µg/mL). On the other hand, sesamin was highly antiproliferative against HCC 1395, with an IC_50_ value of 3.39 ± 1.0 µg/mL. The selectivity index for the human breast cancer cell line, HCC 1395, was 39.97, showing that it was highly selective. However, it was less selective against the human prostate cancer cell line DU 145, with an SI value of 1.18. This study is in agreement with reports that sesamin has a strong chemopreventative activity against mammary tumors in rats [52]. Sesamin was also reported to suppress cell cycle progression and angiogenesis in different mammary cancer models [53,54]

A variable antiproliferative activity was observed for 8-acetonyldihydrochelerythrine (**4**), depending on the cell line used. The CC_50_ value with relation to Vero E6 was 47.83 ± 1.15 µg/mL, and the IC_50_ value for DU 145 was 66.82 ± 0.58 µg/mL, while the IC_50_ value for HCC 1395 was 9.99 ± 0.6 µg/mL. 8-Acetonyldihydrochelerythrine was highly selective against HCC 1395, with the SI value of 4.79, and was less selective against DU 145, with which the SI value was 0.72. This is the first report on the antiproliferative activity of 8-acetonyldihydrochelerythrine. However, the antiproliferative activities of related phenanthridine alkaloids have been reported, which illustrates that this class of compounds enact significant antitumor activities in cancer cells [55,56].

The antiproliferation effect against Vero E6 was high, with a CC_50_ value of 2.77 ± 0.12 µg/mL being recorded when arnottianamide (**5**) was applied to the cells. There was also evidence of the moderate antiproliferative activity of this compound against both HCC 1395 and DU 145, with which the IC_50_ values were 38.34 ± 0.1 µg/mL and 84.31 ± 0.64 µg/mL, respectively. From the SI values, it was evident that arnottianamide is less selective against both HCC 1395 (with the SI value of 0.07) and DU 145 (with the SI value of 0.03). The lack of specificity of this compound in different cancer cell lines has been reported [57,58]. 

Other alkaloids that occur in the *Zanthoxylum* species may also be important for the anticancer effects of their extracts. In fact, several compounds that are alkaloids are now in routine use as anticancer drugs, such as taxol [59]. In this study, 10-methoxycanthin-6-one (**6**) indicated a moderate activity against Vero E6, with a CC_50_ value of 53.95 ± 0.38 µg/mL, and an antiproliferative activity against HCC 1395 (IC_50_ value of 14.70 ± 0.5 µg/mL) and high antiproliferative activity against DU 145 (IC_50_ value of 1.58 ± 0.00 µg/mL). 10-Methoxycanthin-6-one was highly selective against the two cancer cell lines, with an SI values in relation to HCC 1395 of 3.67, while that for DU 145 was 34.15 (Table 2). This is the first report on the antiproliferative activity of 10-methoxycanthin-6-one. Another alkaloid with high anticancer activity is canthin-6-one (**7**), which, based on the median inhibitory concentration (IC_50_/CC _50_) values, was found to have a moderate activity against Vero E6 (with a CC_50_ of 41.81 ± 0.64 µg/mL) and an activity against both HCC 1395 and DU 145 (IC_50_ values of 8.12 ± 0.6 µg/mL and 9.43 ± 0.01 µg/mL, respectively). The SI values indicated that the compound was highly selective against both HCC 1395 and DU 145, with SI values of 5.15 and 4.43, respectively. A report by Dai et al. [60] indicates that canthin-6-one has a broad anticancer activity, which has made the compound useful as an anticancer agent [61]. Additionally, canthin-6-one isolated from the stem bark of *Z. paracanthum* exhibited a high antiproliferative activity against leukemia cancer cell lines [11].

8-Oxochelerythrine (**8**) is shown here for the first time to be toxic against HCC 1395 (with an IC_50_ value of 14.09 ± 0.3 µg/mL) and moderately toxic against DU 145 (with an IC_50_ of 63.41 ± 1.10 µg/mL). Conversely, it is inactive against the normal cell line, the Vero E6 cell line (a CC_50_ value of 135.32 ± 0.12 µg/mL). This compound was highly selective against the human breast cancer cell line, HCC 1395, with an SI value of 9.60, while that of the human prostate cancer cell line, DU 145, was 2.13. This is the first report on the antiproliferative activity of 8-oxochelerythrine; however, the studies on the antiproliferative activity of some of the other benzophenanthridine alkaloids, already mentioned in this study, authenticate the present report. The percentage inhibitions of the tested samples against HCC 1395, DU 145 and Vero E6 are summarized in Figure 2, Figure 3 and Figure 4, respectively. The CC_50_/IC_50_ values and the selectivity indexes for different compounds against selected cell lines are presented in Table 2.

## 3. Materials and Methods

### 3.1. General

Column chromatography was done on silica gel 60 (70 230 mesh), and thin layer chromatography (TLC) on silica gel 60 F254, Merck). The NMR spectra were recorded using Bruker Avance 500 MHz spectrometers. COSY, HSQC, NOESY and HMBC spectra were obtained using standard Bruker software. Chemical shifts were measured in ppm in δ values relative to the internal standard tetramethyl silane (TMS). Omacilin and fluconazole were bought from Sigma-Aldrich (USA). The cell lines used in the cytotoxicity tests were obtained from Manassas, VA, USA. Doxorubicin was obtained from Johnson & Johnson, USA. The microorganisms used in this study were provided by the Centre for Microbiology Research (CMR), Kenya Medical Research Institute (KEMRI).

### 3.2. Plant Materials 

The roots of *Z. paracanthum* were collected from Mrima Hills, Kwale County in Kenya (GPS coordinates 4°29′01.6″ S and 39°15′30.8″ E) in April 2018. The plant was positively identified by Ms Magrate Kaigongi, a plant taxonomist at the Kenya Forestry Research Institute, and a voucher specimen was deposited at the University Nairobi Herbarium (NAI) under voucher specimen number MK14/2018. The plant samples (2 kg) were washed in running water to remove soil, chopped into small pieces (~2 cm) and air dried for three weeks before being ground into fine material using an electrically powered grinder.

### 3.3. Extraction, Isolation and Elucidation of Compounds 

The ground root bark (960 g) of *Z. paracanthum* was exhaustively extracted for 72 h with dichloromethane:Methanol (CH_2_Cl_2_:CH_3_OH) (1:1). The resultant extract was filtered and the solvent removed in vacuo to give 128 g of the crude extract. A portion of this extract (77 g) was partitioned between CH_2_Cl_2_ and water, followed by ethyl acetate (EtOAc) and water. Removal of the organic solvents yielded 45 g of CH_2_Cl_2_ and 3 g of EtOAc extracts. The CH_2_Cl_2_ extract (45 g) was subjected to column chromatography on silica gel (700 g) and eluted with *n*-hexane containing increasing amounts of EtOAc (1%, 2%, 4%, 6%, 8%, 10%, 15%, 20%, 30%, …90% and 100%) and then with EtOAc containing increasing amounts of CH_3_OH (5%, 10%, 20%, …100%). A total of 220 fractions, each 400 mL, were collected and combined on the basis of thin layer chromatography (TLC) analysis. The fractions eluted with 1% to 3% EtOAc in *n*-hexane were not followed further. The fractions eluted with 4% EtOAc in *n*-hexane were combined and further purified by column chromatography on Sephadex LH-20 (eluent: CH_2_Cl_2_/CH_3_OH, 1:1) to yield compound **1** (50 mg). The fractions eluted at 6–10% EtOAc in *n*-hexane were combined, then separated by column chromatography over silica gel eluting with *n*-hexane (EtOAc, 4:1) to yield compound **2** (100 mg), compound **3** (320 mg) and compound **4** (101 mg). The fractions obtained at 12% EtOAc were combined and crystallized to give compound **5** (42 mg). The eluents obtained with 15–70% EtOAc in *n*-hexane were combined and purified on Sephadex (eluent: CH_2_Cl_2_/CH_3_OH, 1:1) to afford compound **6** (68 mg), compound **7** (10 g) and compound **8** (54 mg). The EtOAc extract (3 g) was not followed further. The structures of the purified compounds were determined using nuclear magnetic resonance (NMR), spectroscopy, mass spectrometry and comparison with the literature.

### 3.4. In Vitro Antimicrobial Testing

All the isolated compounds and the root bark extract of *Z. paracanthum* were tested for antimicrobial activity against an un-typed isolate of methicillin resistant *Staphylococcus aureus* (MRSA), *Escherichia coli* (ATCC 25922), *Staphylococcus aureus* (ATCC 29213) and *Candida albicans* (ATCC 10231). 

A broth dilution with two-fold serial dilutions of each sample was carried out ranging from 1000 µg/mL to 0.011 µg/mL in 96 micro well plates, to determine the MIC. All the dilutions were carried out in triplicate. One milliliter of 24 h and 72 h culture, for bacteria and fungi respectively (≅10^6^ CFU/mL), adjusted to McFarland turbidity, was added to each dilution and incubated at 37 °C for 24 h for bacteria and 30 °C for 72 h for fungi. The lowest dilution with undetectable bacterial growth was recorded as the MIC. A lack of growth of the microorganisms was validated by the absence of turbidity after inoculating into agar followed by incubations for 24 and 72 h, for bacteria and fungi, respectively [62].

### 3.5. In Vitro Antiproliferative Activity Testing

All eight of the isolated compounds were tested against three cell lines, namely, human breast cancer cell line (HCC 1395), human prostate cancer cell line (DU 145) and normal cell line (Vero E6). Seven concentrations of each compound in dimethyl sulfoxide (DMSO) (100, 33.33, 11.11, 3.70, 1.23, 0.41 and 0.14 μg/mL) were used in triplicate. The chemotherapeutic drug doxorubicin was used as positive control in the aforementioned concentrations, while cells in minimum essential media were used as negative control. Methyl-tetrazolium (MTT) assay was used to determine the antiproliferative activity.

The cells preserved in liquid nitrogen in vials were removed and thawed in a water bath at 37 °C. This was followed by centrifuging the vial contents and transferring the supernatant into a growth minimum essential media (MEM) enriched with 10 v/v of Fetal Bovine Serum (FBS), 1 v/v antibiotic and 1 v/v L-Glutamine in a T75 culture flask, which was incubated for 48 h at 37°C in 5 v/v CO_2_ to reach confluence level. After cells for all the cell lines attained confluence, they were cleaned with saline phosphate buffer and harvested by trypsinization. The viable cells were calculated by determining cell density using the Trypan blue exclusion method in a haemocytometer. An aliquot of 100 µL containing 2.0 × 10^4^ cells/mL suspension was seeded into a 96-well plate and incubated for 24 h at 37 °C in v/v CO_2_. Fifteen microliters of test plant extracts were added at seven different concentrations to each serial dilution, from row H to B in a 96-well plate. Row A, containing cells and medium, only served as the negative control, while row H had the highest sample concentration (100 µg/mL). Doxorubicin was used as the positive control. The experiment was done in triplicates and incubated for 48 h. Thereafter, 10 µL of MTT dye (5 mg/mL) was added into each well and the plates were incubated at 37 °C for 2 h in 5% CO_2_. The enzyme mitochondrial dehydrogenase, a biomarker of live cells, is known to interact with and reduce MTT dye to an insoluble formazan, which is purple in color. The amount of formazan formed is directly proportional to the number of live cells. Formazan formation was solubilized with 50 µL of DMSO and confirmed using an inverted light microscope. The antiproliferative activity of the test samples on the cancer and normal cells was expressed in IC_50_ values (the sample concentration which killed 50% of the cancer cells) and CC_50_ values (concentration of sample that exerted lethal effects on 50% of the normal cells) [63].

### 3.6. Data Analysis

#### 3.6.1. Calculation of Percentage Cytotoxicity

Cytotoxicity was calculated using the following formula (Equation (1)) [64]:

% Cell viability = (A_T_ − A_B_) / (A_C_ – A_B_) × 100 (1)

where A_T_^=^ Absorbance of treated cells (drug), A_B_^=^ Absorbance of blank (only media) and A_C_^=^ Absorbance of control (untreated) (Equation (2)).

%Cytotoxicity = 100 − % cell viability(2)

The Finney’s Probit analysis using BioStat version 6.7 was used to calculate the CC _50_ and IC_50_ values. The values were then analyzed using a Tukey’s test to determine statistical significance at a probability level of 0.05.

#### 3.6.2. Selectivity Index Determination

Selectivity index (SI), which shows the capability of a treatment to selectively exert toxicity towards cancerous cells and spare the normal cells, was determined using (Equation (3)) [65]:

(3) $$\mathrm{SI}=\frac{{\mathrm{CC}}_{50}}{{\mathrm{IC}}_{50}}$$

where CC_50_ is concentration of the compound that killed 50% of the normal cells, and IC_50_ is concentration of the compound killed 50% of cancerous cells. 

## 4. Conclusions

In this study, eight compounds (myristic acid (**1**), stigmasterol (**2**), 8-acetonyldihydrochelerythrine (**4**), arnottianamide (**5**), 10-methoxycanthin-6-one (**6**) and 8-oxochelerythrine (**8**)) were isolated and characterized from the root bark extract of *Z. paracanthum* for the first time, adding new information to better define the phytochemistry of this plant. Both the root bark extract and the purified compounds were tested using in vitro-based antimicrobial and antiproliferation cancer assays. All other previous investigations have examined only the stem extracts of this species. The present study thus supports and substantiates the work of others, as it highlights the value of analyzing different plant parts for bioactivity. Other studies of the chemistries and bioactivities of the leaf extracts of this species should be carried out, as leaves are a more renewable source of material for pharmaceutical application, compared to both stems and roots. This study provides scientific evidence for the popularity of root extracts of *Z. paracanthum* as a traditional plant medicine, which is taken for microbial infections and/or cancer-related ailments in locations where the plant is found.

## Figures and Tables

**Table 1:** Antimicrobial minimal inhibition concentration (MIC) values (µg/mL) of the tested samples.

Sample Name	Microbial Organism			
	MRSA	*S. aureus*	*E. coli*	*C. albicans*
myristic acid (**1**)	>1000	>1000	>1000	>1000
stigmasterol (**2**)	>1000	62.50	15.63	>1000
sesamin (**3**)	>1000	500	>1000	>1000
8- acetonyldihydrochelerythrine (**4**)	31.25	15.63	15.63	62.50
arnottianamide (**5**)	>1000	>1000	>1000	>1000
10-methoxycanthin-6-one (**6**)	3.91	1.95	3.91	7.81
canthin-6-one (**7**)	0.98	0.49	1.95	3.91
8-oxochelerythrine (**8**)	62.50	7.81	3.91	15.63
*Z. paracanthum* root bark extract	3.91	0.98	1.95	7.81
Omacilin	0.98	0.49	0.98	-
Fluconazole	-	-	-	1.95

MIC—Minimal inhibition concentration. MRSA—methicillin resistant *Staphylococcus aureus*, *Escherichia coli* (ATCC 25922), *Staphylococcus aureus* (ATCC 29213) and *Candida albicans* (ATCC 10231). MIC values for canthin- 6- one (**7**) against MRSA and *S. aureus* were similar to omacilin (positive control).

**Table 2:** CC_50_/ IC_50_ (µg/mL) and SI values for different samples against selected cell lines.

Sample Names	CC_50_ Normal Cell Line	IC_50_ Cancerous Cell Lines		Selectivity Indexes	
	Vero E6	HCC 1395	DU 145	HCC 1395	DU 145
myristic acid (**1**)	64.86 ± 0.51 ^c^	57.71 ± 1.2 ^a^	80.24 ± 0.12 ^d^	1.12	0.81
stigmasterol (**2**)	123.88 ± 0.00 ^b^	0.42 ± 0.1 ^i^	140.49 ± 1.27 ^a^	294.94	0.88
sesamin (**3**)	135.31 ± 0.12 ^a^	3.39 ± 1.0 ^h^	115.06 ± 0.03 ^b^	39.97	1.18
8-acetonyldihydrochelerythrine (**4**)	47.83 ± 1.15 ^e^	9.99 ± 0.6 ^e^	66.82 ± 0.58 ^e^	4.79	0.72
arnottianamide (**5**)	2.77 ± 0.12 ^h^	38.34 ± 0.1 ^b^	84.31 ± 0.64 ^c^	0.07	0.03
10-methoxycanthin-6-one (**6**)	53.95 ± 0.38 ^d^	14.70 ± 0.5 ^c^	1.58 ± 0.00 ^i^	3.67	34.15
canthin-6-one (**7**)	41.81 ± 0.64 ^f^	8.12 ± 0.6 ^f^	9.43 ± 0.01 ^h^	5.15	4.43
8-oxochelerythrine (**8**)	135.32 ± 0.12 ^a^	14.09 ± 0.3 ^d^	63.41 ± 1.10 ^f^	9.60	2.13
*Z. paracanthum* root bark extract	28.28 ± 0.34 ^g^	7.27 ± 0.0 ^g^	53.21 ± 1.21 ^g^	3.89	0.53
Doxorubicin (Positive control)	0.30 ± 0.12 ^i^	0.21 ± 0.2 ^i^	0.59 ± 0.01 ^i^	1.41	0.51

Vero E6—Normal cell line, HCC 1395—human breast cancer cell line, DU 145—human prostate cancer cell line, SI—selectivity index. Values for CC_50_/ IC_50_ are expressed as Mean ± SEM, values sharing a superscript letter in each column are not significantly different from each other (*p* ≥ 0.05).

## Supplementary Materials

The following are available online at https://www.mdpi.com/2223-7747/9/7/920/s1, Table S1: ^1^H, ^13^C NMR data and HMBC correlations for myristic acid (1), Table S2: ^1^H, ^13^C NMR data and HMBC correlations for stigmasterol (2), Table S3: ^1^H, ^13^C NMR data and HMBC correlations for sesamin (3). Table S4: ^1^H, ^13^C NMR data and HMBC correlations for 8-acetonyldihydrochelerythrine (4). Table S5: ^1^H, ^13^C NMR data and HMBC correlations for arnottianamide (5), Table S6: ^1^H, ^13^C NMR data and HMBC correlations for 10-methoxycanthin-6-one (6), Table S7: ^1^H, ^13^C NMR data and HMBC correlations for canthin-6-one (7), Table S8: ^1^H, ^13^C NMR data and HMBC correlations for 8-oxochelerythrine (8), Figure S1: The ^1^H NMR spectrum of myristic acid (1) observed at 500 MHz for CDCl_3_ solution at 25 °C. Assignment is given in Table S1, Figure S2: The ^13^C NMR spectrum of myristic acid (1) observed at 125 MHz for CDCl_3_ solution at 25 °C. Assignment is given in Table S1, Figure S3: The ^1^H-^1^H COSY spectrum of myristic acid (1) observed at 500 MHz for CDCl_3_ solution at 25 °C, Figure S4: The ^1^H-^13^C HSQC NMR spectrum of myristic acid (1) observed at 500 and 125 MHz for CDCl_3_ solution at 25 °C, Figure S5: The ^1^H-^13^C HMBC NMR spectrum of myristic acid (1) observed at 500 and 125 MHz for CDCl_3_ solution at 25 °C. Assignment is given in Table S1, Figure S6: The ESIMS spectrum for myristic acid (1), Figure S7: The ^1^H NMR spectrum of stigmasterol (2) observed at 500 MHz for CDCl_3_ solution at 25 °C. Assignment is given in Table S2, Figure S8: The ^13^C NMR spectrum of stigmasterol (2) observed at 125 MHz for CDCl_3_ solution at 25 °C. Assignment is given in Table S2, Figure S9: The ^1^H-^1^H COSY spectrum of stigmasterol (2) observed at 500 MHz for CDCl_3_ solution at 25 °C, Figure S10: The ^1^H-^13^C HSQC NMR spectrum of stigmasterol (2) observed at 500 and 125 MHz for CDCl_3_ solution at 25 °C, Figure S11: The ^1^H-^13^C HMBC NMR spectrum of stigmasterol (2) observed at 500 and 125 MHz for CDCl_3_ solution at 25 °C. Assignment is given in Table S2, Figure S12: The ESIMS spectrum of stigmasterol (2), Figure S13: The ^1^H NMR spectrum of sesamin (3) observed at 500 MHz for CDCl_3_ solution at 25 °C. Assignment is given in Table S3, Figure S14: The ^13^C NMR spectrum of sesamin (3) observed at 125 MHz for CDCl_3_ solution at 25 °C. Assignment is given in Table 1. Assignment is given in Table S3, Figure S15: The ^1^H-^1^H COSY spectrum of sesamin (3) observed at 500 MHz for CDCl_3_ solution at 25, Figure S16: The ^1^H-^13^C HSQC NMR spectrum of sesamin (3) observed at 500 and 125 MHz for CDCl_3_ solution at 25 °C, Figure S17: The ^1^H-^13^C HMBC NMR spectrum of sesamin (3) observed at 500 and 125 MHz for CDCl_3_ solution at 25 °C. Assignment is given in Table S3, Figure S18: The ESIMS spectrum of sesamin (3), Figure S19: The ^1^H NMR spectrum of 8-acetonyldihydrochelerythrine (4) observed at 500 MHz for CDCl_3_ solution at 25 °C. Assignment is given in Table S4, Figure S20: The ^13^C NMR spectrum of 8-acetonyldihydrochelerythrine (4) observed at 125 MHz for CDCl_3_ solution at 25 °C. Assignment is given in Table S4, Figure S21: The ^1^H-^1^H COSY spectrum of 8-acetonyldihydrochelerythrine (4) observed at 500 MHz for CDCl_3_ solution at 25 °C, Figure S22: The ^1^H-^13^C HSQC NMR spectrum of 8-acetonyldihydrochelerythrine (4) observed at 500 and 125 MHz for CDCl_3_ solution at 25 °C, Figure S23: The ^1^H-^13^C HMBC NMR spectrum of 8-acetonyldihydrochelerythrine (4) observed at 500 and 125 MHz for CDCl_3_ solution at 25 °C. Assignment is given in Table S4, Figure S25: The ^1^H NMR spectrum of arnottianamide (5) observed at 500 MHz for CD_2_Cl_2_ solution at 25 °C. Assignment is given in Table S5, Figure S26: The ^13^C NMR spectrum of arnottianamide (5) observed at 125 MHz for CD_2_Cl_2_ solution at 25 °C. Assignment is given in Table S5, Figure S27: The ^1^H-^1^H COSY spectrum of arnottianamide (5) observed at 500 MHz for CD_2_Cl_2_ solution at 25 °C, Figure S28: The ^1^H-^13^C HSQC NMR spectrum of arnottianamide (5) observed at 500 and 125 MHz for CD_2_Cl_2_ solution at 25 °C, Figure S29: The ^1^H-^13^C HMBC NMR spectrum of arnottianamide (5) observed at 500 and 125 MHz for CD_2_Cl_2_ solution at 25 °C. Assignment is given in Table S5, Figure S30: The ESIMS spectrum for arnottianamide (5), Figure S31: The ^1^H NMR spectrum of 10-methoxycanthn-6- one (6) observed at 500 MHz for CD_2_Cl_2_ solution at 25 °C. Assignment is given in Table S6, Figure S32: The ^13^C NMR spectrum of 10-methoxycanthn-6- one (6) observed at 125 MHz for CD_2_Cl_2_ solution at 25 °C. Assignment is given in Table S6, Figure S33: The ^1^H-^1^H COSY spectrum of 10-methoxycanthn-6- one (6) observed at 500 MHz for CD_2_Cl_2_ solution at 25 °C, Figure S34: The ^1^H-^13^C HSQC NMR spectrum of 10-methoxycanthn-6- one (6) observed at 500 and 125 MHz for CD_2_Cl_2_ solution at 25 °C, Figure S35: The ^1^H-^13^C HMBC NMR spectrum of 10-methoxycanthn-6- one (6) observed at 500 and 125 MHz for CD_2_Cl_2_ solution at 25 °C. Assignment is given in Table S6, Figure S36: The ESIMS spectrum for 10-methoxycanthin- 6- one (6), Figure S37: The ^1^H NMR spectrum of canthin-6-one (7) observed at 500 MHz for CD_2_Cl_2_ solution at 25 °C. Assignment is given in Table S7, Figure S38: The ^13^C NMR spectrum of canthin-6-one (7) observed at 125 MHz for CD_2_Cl_2_ solution at 25 °C. Assignment is given in Table S7, Figure S39: The ^1^H-^1^H COSY spectrum of canthin-6-one (7) observed at 500 MHz for CD_2_Cl_2_ solution at 25 °C, Figure S40: The ^1^H-^13^C HSQC NMR spectrum of canthin-6-one (7) observed at 500 and 125 MHz for CD_2_Cl_2_ solution at 25 °C, Figure S41: The ^1^H-^13^C HMBC NMR spectrum of canthin-6-one (7) observed at 500 and 125 MHz for CD_2_Cl_2_ solution at 25 °C. Assignment is given in Table S7, Figure S42: The ESIMS spectrum of canthin- 6- one (7), Figure S43: The ^1^H NMR spectrum of 8-oxochelerythrine (8) observed at 500 MHz for CDCl_3_ solution at 25 °C. Assignment is given in Table S8, Figure S44: The ^13^C NMR spectrum of 8-oxochelerythrine (8) observed at 125 MHz for CDCl_3_ solution at 25 °C. Assignment is given in Table S8, Figure S45: The ^1^H-^1^H COSY spectrum of 8-oxochelerythrine (8) observed at 500 MHz for CDCl_3_ solution at 25 °C, Figure S46: The ^1^H-^13^C HSQC NMR spectrum of 8-oxochelerythrine (8) observed at 500 and 125 MHz for CDCl_3_ solution at 25 °C, Figure S47: The ^1^H-^13^C HMBC NMR spectrum of 8-oxochelerythrine (8) observed at 500 and 125 MHz for CDCl_3_ solution at 25 °C. Assignment is given in Table S8, Figure S48: The ESIMS spectrum for 8-oxocheerythrine (8).

## References

[1] Ang-Lee M.K., Moss J., Yuan C.S. (2001). Herbal medicines and perioperative care. JAMA.

[2] Ochwang’i D.O., Kimwele C.N., Oduma J.A., Gathumbi P.K., Mbaria J.M., Kiama S.G. (2014). Medicinal plants used in treatment and management of cancer in Kakamega County, Kenya. J. Ethnopharmacol..

[3] Korir A., Okerosi N., Ronoh V., Mutuma G., Parkin M. (2015). Incidence of cancer in Nairobi, Kenya (2004–2008). Int. J. Cancer..

[4] Macharia L.W., Mureithi M.W., Anzala O. (2019). Cancer in Kenya: Types and infection-attributable. Data from two National referral hospitals. AAS Open Res..

[5] Colditz G.A., Wolin K.Y., Gehlert S. (2012). Applying what we know to accelerate cancer prevention. Sci. Transl. Med..

[6] Amin A., Gali-Muhtasib H., Ocker M., Schneider-Stock R. (2009). Overview of major classes of plant-derived anticancer drugs. Int. J. Biomed. Sci. IJBS.

[7] Shah U., Shah R., Acharya S., Acharya N. (2013). Novel anticancer agents from plant sources. Chin. J. Nat. Med..

[8] Saunders F.R., Wallace H.M. (2010). On the natural chemoprevention of cancer. Plant. Physiol. Biochem..

[9] Kokwaro J.O. (1976). Medicinal Plants of East Africa.

[10] Beentje H., Adamson J., Bhanderi D. (1994). Kenya Trees, Shrubs, and Lianas.

[11] Omosa L.K., Mbogo G.M., Korir E., Omole R., Seo E.J., Yenesew A., Heydenreich M., Midiwo J.O., Efferth T. (2019). Cytotoxicity of fagaramide derivative and canthin-6-one from *Zanthoxylum* (Rutaceae) species against multidrug resistant leukemia cells. Nat. Prod. Res..

[12] Lyimo M., Temu R.P.C., Mugula J.K. (2003). Identification and nutrient composition of indigenous vegetables of Tanzania. Plant Foods Hum. Nutr..

[13] Samita F.N., Sandjo L.P., Ndiege I.O., Hassanali A., Lwande W. (2013). Zanthoxoaporphines A–C: Three new larvicidal dibenzo [de, g] quinolin-7-one alkaloids from *Zanthoxylum paracanthum* (Rutaceae). Beilstein J. Org. Chem..

[14] Islam A., Sayeed A., Bhuiyan M.S.A., Mosaddik M.A., Islam M.A.U., Khan G.R.M.A.M. (2001). Antimicrobial activity and cytotoxicity of *Zanthoxylum budrunga*. Fitoterapia.

[15] Chrian M., Erasto P., Otieno N.J. (2011). Antimycobacterial activity and cytotoxicity effect of extracts of *Hallea rubrostipulata* and *Zanthoxylum chalybeum*. Spat DD.

[16] Misra L.N., Wouatsa N.A.V., Kumar S., Kumar R.V., Tchoumbougnang F. (2013). Antibacterial, cytotoxic activities and chemical composition of fruits of two Cameroonian *Zanthoxylum* species. J. Ethnopharmacol..

[17] Yaouba S. (2018). Phytochemical Investigation of Selected Plants in the Families Anacardiaceae and Asteraceae for Bioactive Principles. Ph.D. Thesis.

[18] Ross S.A., Krishnaven K., Radwan M.M., Takamatsu S., Burandt C.L. (2008). Constituents of *Zanthoxylum flavum* and their antioxidant and antimalarial activities. Nat. Prod. Commun..

[19] Omosa L.K., Okemwa E.K. (2017). Antiplasmodial Activities of the Stem bark Extract and Compounds of *Zanthoxylum gilletii* (De wild) PG Waterman. Pharmacogn. Commun..

[20] Adesina S.K., Reisch J. (1988). Arnottianamide and other constituents of *Zanthoxylum gillettii* root. J. Nat. Prod..

[21] Ferreira M.E., De Arias A.R., De Ortiz S.T., Inchausti A., Nakayama H., Thouvenel C., Hocquemiller R., Fournet A. (2002). Leishmanicidal activity of two canthin-6-one alkaloids, two major constituents of *Zanthoxylum chiloperone* var. *angustifolium*. J. Ethnopharmacol..

[22] Koul S., Razdan T.K., Andotra C.S., Kalla A.K., Koul S., Taneja S.C. (2002). Benzophenanthridine alkaloids from *Corydalis flabellata*. Planta Med..

[23] Kaigongi M.M., Dossaji S.F., Nguta J.M., Lukhoba C.W., Musila F.M. (2014). Antimicrobial activity, toxicity and phytochemical screening of four medicinal plants traditionally used in Msambweni district, Kenya. J. Biol. Agric. Healthc..

[24] Gaya C.H., Kawaka J.F., Muchugi A., Ngeranwa J.J. (2013). Variation of alkaloids in the Kenyan *Zanthoxylum gilletii* (De Wild Waterman). Afr. J. Plant Sci..

[25] Buyinza D. (2012). Phytochemical Investigation of Zanthoxylum Holstzianum for Antimicrobial Principles. Ph.D. Thesis.

[26] Kitahara T., Koyama N., Matsuda J., Aoyama Y., Hirakata Y., Kamihira S., Kohno S., Nakashima M., Sasaki H. (2004). Antimicrobial activity of saturated fatty acids and fatty amines against methicillin-resistant *Staphylococcus aureus*. Biol. Pharm. Bull..

[27] Laggoune S., Boutaghane N., Kabouche A., Kabouche Z., Ait-Kaki Z., Ait-Kaki B. (2008). Components and antimicrobial activity of *Lamium amplexicaule* from Algeria. Chem. Nat. Compd..

[28] Chakraborty S., Tiedemann A.V., Teng P.S. (2000). Climate change: Potential impact on plant diseases. Environ. Pollut..

[29] Zhou J., Sun M., Wang H., Yan X., Yao M., Zhu C., Wang L.J., Zhou J.C., Liu B.L. (2004). Studies on Antibacteria and Antioxidant Properies of Sesamin. Food Sci..

[30] Enright M.C., Robinson D.A., Randle G., Feil E.J., Grundmann H., Spratt B.G. (2002). The evolutionary history of methicillin-resistant *Staphylococcus aureus* (MRSA). Proc. Natl. Acad. Sci. USA.

[31] Tantapakul C., Phakhodee W., Ritthiwigrom T., Yossathera K., Deachathai S., Laphookhieo S. (2012). Antibacterial compounds from *Zanthoxylum rhetsa*. Arch. Pharm. Res..

[32] Queiroz E.F., Hay A.E., Chaaib F., van Diemen D., Diallo D., Hostettmann K. (2006). New and bioactive aromatic compounds from *Zanthoxylum zanthoxyloides*. Planta Med..

[33] Cabral V., Luo X., Junqueira E., Costa S.S., Mulhovo S., Duarte A., Couto I., Viveiros M., Ferreira M.J. (2015). Enhancing activity of antibiotics against Staphylococcus aureus: *Zanthoxylum capense* constituents and derivatives. Phytomedicine.

[34] Zhao F., Dai J.K., Liu D., Wang S.J., Wang J.R. (2016). Synthesis and evaluation of ester derivatives of 10-hydroxycanthin-6-one as potential antimicrobial agents. Molecules.

[35] Li N., Liu D., Dai J.K., Wang J.Y., Wang J.R. (2019). Synthesis and In Vitro Antibacterial Activity of Quaternized 10-Methoxycanthin-6-one Derivatives. Molecules.

[36] Soriano-Agatón F., Lagoutte D., Poupon E., Roblot F., Fournet A., Gantier J.C., Hocquemiller R. (2005). Extraction, hemisynthesis, and synthesis of canthin-6-one analogues. Evaluation of their antifungal activities. J. Nat. Prod..

[37] Gazoni V.F., Balogun S.O., Arunachalam K., Oliveira D.M., Cechinel Filho V., Lima S.R., Colodel E.M., Soares I.M., Ascêncio S.D., de Oliveira Martins D.T. (2018). Assessment of toxicity and differential antimicrobial activity of methanol extract of rhizome of *Simaba ferruginea* A. St.-Hil. and its isolate canthin-6-one. J. Ethnopharmacol..

[38] Cesari I., Grisoli P., Paolillo M., Milanese C., Massolini G., Brusotti G. (2015). Isolation and characterization of the alkaloid Nitidine responsible for the traditional use of *Phyllanthus muellerianus* (Kuntze) Excell stem bark against bacterial infections. J. Pharm. Biomed. Anal..

[39] Tavares L de C., Zanon G., Weber A.D., Neto A.T., Mostardeiro C., Da Cruz I.B.M., Oliveira R.M., Ilha V., Dalcol I.I., Morel A.F. (2014). Structure-activity relationship of benzophenanthridine alkaloids from *Zanthoxylum rhoifolium* having antimicrobial activity. PLoS ONE.

[40] Boik J. (2001). Natural Compounds in Cancer Therapy.

[41] Badisa R.B., Darling-Reed S.F., Joseph P., Cooperwood J.S., Latinwo L.M., Goodman C.B. (2009). Selective cytotoxic activities of two novel synthetic drugs on human breast carcinoma MCF-7 cells. Anticancer Res..

[42] Menezes C., Valério E., Dias E. (2013). The kidney Vero-E6 cell line: A suitable model to study the toxicity of microcystins. New Sights into Toxicity and Drug Testing.

[43] Matskevich A.A., Jung J.S., Schümann M., Cascallo M., Moelling K. (2009). Vero cells as a model to study the effects of adenoviral gene delivery vectors on the RNAi system in context of viral infection. J. Innate Immun..

[44] Efferth T., Koch E. (2011). Complex interactions between phytochemicals. The multi-target therapeutic concept of phytotherapy. Curr. Drug Targets.

[45] Talib W.H. (2011). Anticancer and antimicrobial potential of plant-derived natural products. Phytochemicals: Bioactivities and Impact on Health.

[46] Singh T.D., Meitei H.T., Sharma A.L., Robinson A., Singh L.S., Singh T.R. (2015). Anticancer properties and enhancement of therapeutic potential of cisplatin by leaf extract of *Zanthoxylum armatum* DC. Biol. Res..

[47] Wongtangtintharn S., Oku H., Iwasaki H., Toda T. (2004). Effect of branched-chain fatty acids on fatty acid biosynthesis of human breast cancer cells. J. Nutr. Sci. Vitaminol (Tokyo).

[48] Frankfurt O.S., Krishan A. (2003). Apoptosis-based drug screening and detection of selective toxicity to cancer cells. Anticancer Drugs.

[49] Ghosh T., Maity T.K., Singh J. (2011). Evaluation of antitumor activity of stigmasterol, a constituent isolated from *Bacopa monnieri* Linn aerial parts against Ehrlich Ascites Carcinoma in mice. Orient. Pharm. Exp. Med..

[50] Fiorentino A., DellaGreca M., D’Abrosca B., Oriano P., Golino A., Izzo A., Zarrelli A., Monaco P. (2007). Lignans, neolignans and sesquilignans from Cestrum parqui l’Her. Biochem. Syst. Ecol..

[51] Cutillo F., DellaGreca M., Gionti M., Previtera L., Zarrelli A. (2006). Phenols and lignans from *Chenopodium album*. Phytochem. Anal. Int. J. Plant Chem. Biochem. Tech..

[52] Hirose N., Doi F., Ueki T., Akazawa K., Chijiiwa K., Sugano M., Akimoto K., Shimizu S., Yamada H. (1992). Suppressive effect of sesamin against 7, 12-dimethylbenz [a]-anthracene induced rat mammary carcinogenesis. Anticancer Res..

[53] Lee C.C., Liu K.J., Wu Y.C., Lin S.J., Chang C.C., Huang T.S. (2011). Sesamin inhibits macrophage-induced vascular endothelial growth factor and matrix metalloproteinase-9 expression and proangiogenic activity in breast cancer cells. Inflammation.

[54] Yokota T., Matsuzaki Y., Koyama M., Hitomi T., Kawanaka M., Enoki-Konishi M., Okuyama Y., Takayasu J., Nishino H., Nishikawa A. (2007). Sesamin, a lignan of sesame, down-regulates cyclin D1 protein expression in human tumor cells. Cancer Sci..

[55] Lamoral-Theys D., Andolfi A., Van Goietsenoven G., Cimmino A., Le Calvé B., Wauthoz N., Mégalizzi V., Gras T., Bruyère C., Dubois J. (2009). Lycorine, the main phenanthridine Amaryllidaceae alkaloid, exhibits significant antitumor activity in cancer cells that display resistance to proapoptotic stimuli: An investigation of structure− activity relationship and mechanistic insight. J. Med. Chem..

[56] Tsukamoto H., Kondo S., Mukudai Y., Nagumo T., Yasuda A., Kurihara Y., Kamatani T., Shintani S. (2011). Evaluation of anticancer activities of benzo [c] phenanthridine alkaloid sanguinarine in oral squamous cell carcinoma cell line. Anticancer Res..

[57] Chang Y.C., Hsieh P.W., Chang F.R., Wu R.R., Liaw C.C., Lee K.H., Wu Y.C. (2003). Two new protopines argemexicaines A and B and the anti-HIV alkaloid 6-acetonyldihydrochelerythrine from formosan Argemone mexicana. Planta Med..

[58] Sreelekha M., Anto N.P., Anto R.J., Shafi P.M. (2014). Cytotoxicity of 6-acetonyldihydro-chelerythrin, arnottianamide and 6-(2-hydoxypropyl)-dihydrochelerythrine towards human cancer cell lines. Indian J. Chem..

[59] Oberlies N.H., Kroll D.J. (2004). Camptothecin and taxol: Historic achievements in natural products research. J. Nat. Prod..

[60] Dai J., Li N., Wang J., Schneider U. (2016). Fruitful decades for canthin-6-ones from 1952 to 2015: Biosynthesis, chemistry, and biological activities. Molecules.

[61] Murakami C., Fukamiya N., Tamura S., Okano M., Bastow K.F., Tokuda H., Mukainaka T., Nishino H., Lee K.H. (2004). Multidrug-resistant cancer cell susceptibility to cytotoxic quassinoids, and cancer chemopreventive effects of quassinoids and canthin alkaloids. Bioorg. Med. Chem..

[62] Valgas C., Souza S.M., Smânia E.F., Smânia A. (2007). Screening methods to determine antibacterial activity of natural products. Braz. J. Microbiol..

[63] Mbugua R.W., Njagi E.M., Ngule C.M., Mwitari P. (2019). Gene Expression Mediated Antiproliferative Potential and Safety of Selected Medicinal Plants Against Cancerous and Normal Cells. BioRxiv.

[64] Nemati F., Dehpouri A.A., Eslami B., Mahdavi V., Mirzanejad S. (2013). Cytotoxic properties of some medicinal plant extracts from Mazandaran, Iran. Iran. Red Crescent Med. J..

[65] Wilson A.P. (2000). Cytotoxicity and viability assays. Anim. Cell Cult. Pract. Approach.

