# Supplementary material for: In Vitro Antimicrobial and Antiproliferative Activities of the Root Bark Extract and Isolated Chemical Constituents of Zanthoxylum paracanthum Kokwaro (Rutaceae)
Source: Plants (Basel). 2020 Jul 21;9(7):920. doi: 10.3390/plants9070920 (PMC7412065; doi:10.3390/plants9070920)
Supplement: Supplementary file 1 [file plants-09-00920-s001.pdf]

plants

Supplementary Materials

Contents

1.1. General characteristics of compounds 1-8 2

1.2. Spectroscopic data for compounds 1-8........ 2

1.3. NMR and MS Spectra of the isolated compounds 1-8........ 6

1.3.1. Myristic acid (**1**) 6

1.3.2. Stigmasterol (**2**) 8

1.3.3. Sesamin (**3**) 11

1.3.4. 8-Acetonyldihydrochelerythrine (**4**) 14

1.3.5. Arnottianamide (**5**)........ 18

1.3.6. 10-Methoxycanthn-6- one (**6**) 20

1.3.7. Canthin-6-one (**7**)........ 23

1.3.8. 8-Oxochelerythrine (**8**) 26

References 1

1.1. General characteristics of compounds 1-8

Myristic acid (**1**) [1], a white powder with a molecular formula of $C_{14}H_{28}O_2$, was characterised based on electrospray ionization mass spectrometry (ESIMS)([M-H]$^-$ *m/z* 227). Stigmasterol (**2**) [2] is a white amorphous solid, $C_{29}H_{48}O$, ESIMS ([M-H]$^-$ *m/z* 411). Sesamin(**3**) [3] ($C_{20}H_{18}O_6$, ESIMS ([M-H]$^-$ *m/z* 353)), is a white amorphous solid active under UV (254 nm). 8-Acetonyldihydrochelerythrine (**4**) [4], is a cream powder active under UV (254-366 nm) with ESIMS of ([M+H]$^+$ *m/z* 406), $C_{24}H_{23}NO_5$. Arnottianamide (**5**) [5], is a cream powder that also fluoresced in UV (254-366 nm) with an ESIMS of ([M+H]$^+$ *m/z* 382), $C_{21}H_{19}NO_6$. 10-Methoxycanthn-6- one (**6**) [6] yellow powder with with an ESIMS of ([M+H]$^+$ *m/z* 251), $C_{15}H_{10}N_2O_2$. Canthin-6-one(**7**) [3] orange powder UV active (254-366 nm) hasa n ESIMS of [M+H]$^+$ *m/z* 221, $C_{14}H_8N_2O$. 8-Oxochelerythrine (**8**) [7] white amorphous solid fluoresced in UV (254-366 nm)ESIMS([M+H]$^+$ *m/z* 364)$C_{21}H_{17}NO_5$.

1.2. Spectroscopic data for compounds 1-8

Table S1. ^{1}H, ^{13}C NMR data and HMBC correlations for myristic acid **(1).**

Position	^{13}C NMR (δ_C)	^{1}H NMR (δ_H)	HMBC
1	179.4		
2	33.9	2.35 (t)	C-1, C-3
3	24.7	1.62 (q)	C-2, C-5
4-11	29.6	1.26 (m)	C-3, C-12
12	31.9	12.5 (m)	C-14
13	22.7	1.26 (m)	C-4, C-11
14	14.1	0.88 (t)	C-13, C-12

The NMR assignments and HMBC correlations of myristic acid **(1)** observed at 500MHz dissolved in $CDCL_3$.

Table S2. ^{1}H, ^{13}C NMR data and HMBC correlations for stigmasterol **(2).**

Position	^{13}C NMR (δ_C)	^{1}H NMR (δ_H)	HMBC
1	37.3	1.06(m)	C-3, C-19, C-10
2	31.7	1.88(ddt)	C-10, C-4
3	71.7	3.52(m)	C-4
4	45.8		
5	140.7		
6	129.7	5.35(qd)	C-4, C-7, C-10
7	31.9	1.83(ddq)	C-5, C-9, C-14
8	31.9	2.03 (m)	C-13
9	50.1	0.97 (d)	C-1, C-12
10	36.5		
11	21.2	1.51 (m)	C-8, C-13
12	39.8	2.27 (m);1.99 (m)	C-9, C-14
13	42.3		
14	56.8	1.06(m)	C-16
15	24.3	1.51 (m)	C-7, C-8
16	28.2	1.28 (m)	C-14
17	56.1	1.21	
18	12.0	0.85 (d)	C-12, C-17, C-14
19	18.8	0.97 (d)	C-5
20	40.5	1.99 (m)	C-13, C-23
21	21.1		
22	138.2	5.14 (d)	C-23, C-24, C-20, C-21
23	129.3	5.02 (dd)	C-22
24	51.2		
25	26.1	1.21 (m)	C-23

26	11.9	0.75	
27	29.2	1.31 (m)	C-26, C-29
28	20.5	1.06 (m)	C-29
29	19.1	0.89(m)	C-27

The NMR assignments and HMBC correlations of stigmasterol **(2)** isolated from the root bark of *Zanthoxylum paracanthum* observed at 500 MHz in $CDCl_3$.

Table S3. ^{1}H, ^{13}C NMR data and HMBC correlations for sesamin **(3).**

Position	^{13}C NMR (δ_C)	^{1}H NMR (δ_H)	HMBC
1	54.5	3.06 (m)	C-2, C-1″
2	85.7	4.72 (d, J=4.2)	C-1, C-4, C-2″, C-6″ C-1″
3	O		
4	71.7	4.24 (d, J=4,2);	C-1, C-2
5	54.5	3.06 (m)	C-1, C-1''
6	85.7	4.72(d, J=4.2)	C-4, C-8, C-1″, C-2″
7	O		
8	71.7	3.88 (m)	C-2, C-6
1′	135.6		
2′	106.4	6.88 (s)	C-1', C-4'
3′	147.0		
4′	147.9		
5′	107.9	6.82 (m)	C-3'
6′	119.2	6.84 (m)	C-2'
1″	135.6		
2″	106.4	6.88 (s)	C-4''
3″	147.0		
4″	147.9		
5″	107.9	6.82 (m)	C-1''
6″	119.2	6.84 (m)	C-4''
2x(-O-CH_2-O-)	101.2	5.98 (s)	

The NMR assignments and HMBC correlations of sesamin (**3**) isolated from the root bark of *Zanthoxylum paracanthum* observed at 500MHz dissolved in CD_2Cl_2.

Table S4. ^{1}H, ^{13}C NMR data and HMBC correlations for 8-acetonyldihydrochelerythrine (**4**).

Position	^{13}C NMR (δ_C)	^{1}H NMR (δ_H)	HMBC
1	145.6		
2	152.2		
3	111.7	6.96 (d)	C-1, C-2, C-4a
4	118.8	7.55 (d)	
4a	127.7		
5	131.1		
6	139.1		
7 N-CH_3	42.8	2.64(s)	C-8, C-6
8	55.0	5.05(dd)	C-1, C-6, C-6, C-1′
8a	128.1		
9	119.7	7.71d)	C-5, C-6, C-4a, C-8a
10	124.7	7.51(d)	
10a	123.9		
11	104.3	7.1(s)	C-10a, C-12, C-13, C-14,
12	147.6		
13	148.2		
14	100.6	7.51 (s)	C-12, C-13
14a	127.7		
1′	46.9	2.26(dd),2.64	C-2, C-8, C-8a, C-2′
2′	207.2		

3′	31.0	2.06 (s)	C-1′, C-2′
O-CH_2-O	101.0	6.04 (d)	C-12, C-13
1-OCH_3	60.9	3.96 (s)	C-1
2-OCH_3	55.8	3.92(s)	C-2, C-3

The NMR assignments and HMBC correlations of 8-acetonyldihydrochelerythrine (**4**) isolated from the root bark of *Zanthoxylum paracanthum* observed at 500 MHz dissolved in $CDCl_3$.

Table S5. ^{1}H, ^{13}C NMR data and HMBC correlations for arnottianamide (**5**).

POSITION	^{13}C NMR (δc)	^{1}H NMR (δ$_H$)	HMBC
1	146.9		
2	152.4		
3	113.1	6.98(d)	C-1, C-2
4	119.3	7.60 (d)	C-5 , C-8a
4a	123.9		
5	131.0		
6	140.5		
7	43.44	2.9	C-6, C-8
8	66.1	7.32	
8a	126.0		, C-1, C-5, C-6C-2′
9	117.9	7.52(d)	
10	122.9	7.33(d)	C-5, C-10a, C-11,
10a	122.2		
11	104.2	7.00 (s)	C-10a, C-12, C-13, C-14a
12	147.6		
13	147.9		
14	101.0	7.55	C-5, C-6, C-11, C-12, C-13
14a	125.9		
1′	181.9		
2′	42.8	2.32	
1-OCH_3	60.8	3.81	C-1
2-OCH_3	56.1	3.88	C-1, C-2, C-3
OCH_2O	101.0	6.02	C-12 ,C-13

The NMR assignments and HMBC correlations of arnottianamide (**5**) isolated from the root bark of *Zanthoxylum paracanthum* observed at 500 MHz dissolved in CD_2Cl_2.

Table S6. ^{1}H, ^{13}C NMR data and HMBC correlations for 10-methoxycanthin-6-one (**6**).

Position	^{13}C NMR (δc)	^{1}H NMR (δ$_H$)	HMBC
1	113.6	7.09 (dt)	
2	145.7	8.74	
3	N		
4	139.6	8.02 (d)	C-6, C-11a, C-11b
5	128.4	6.92 (d)	C-3a, C-6, C-7a
6	159.5		
7	N		
8	115.5	7.86 (d)	C-11a, C-11c
9	123.4	7.99 (m)	C-6, C-8, C-10, C-11, C-11b
10	162.5		
11	101.2	8.16 (d)	C-1, C-10, C-11
11a	117.2		
11b	130.2		
11c	132.3		
7a	141.2		
3a	135.5		
-OMe	55.9	4.00 (s)	C-10

The NMR assignments and HMBC correlations of 10-methoxycanthin-6-one (**6**) isolated from the root bark of *Zanthoxylum paracanthum* observed at 500 MHz dissolved in CD_2Cl_2.

Table S7. ^{1}H, ^{13}C NMR data and HMBC correlations for canthin-6-one (**7**).

Position	^{13}C NMR (δc)	^{1}H NMR (δ$_H$)	HMBC
1	116.3	7.90 (d)	C-2, C-7a, C-11b, C-11c,
2	145.5	8.78 (d)	C-1, C-11a, C-11b, C-11c
3	N		
4	139.3	7.98 (d)	C-6, C-7a
5	129.0	6.94 (d)	C-6, C-11b, C-11c
6	159.3		
7	N		
8	117.2	8.6 (dt)	C-9, C-10, C-11, C-11c
9	125.6	7.49 (td)	C-8, C-10, C-11c
10	130.9	7.66 (dd)	
11	122.6	8.03 (m)	C-8, C-10,C-11c
11a	130.3		
11b	135.9		
11c	124.3		
7a	131.95		
3a	139.4		

The NMR assignments and HMBC correlations of canthin-6-one (**7**) isolated from the root bark of *Zanthoxylum paracanthum* observed at 500 MHz dissolved in $CDCl_3$.

Table S8. ^{1}H, ^{13}C NMR data and HMBC correlations for 8-oxochelerythrine (**8**).

Position	^{13}C NMR (δc)	^{1}H NMR (δ$_H$)	HMBC
1	136.5		
2	152.8		
3	103.5	6.55	C-1, C-4a
4	125.1	6.74	C-2, C-5, C-8a
4a	120.1		
5	134.5		
6	135.6		
7	33.1	2.87	C-6, C-8
8	163.8		
8a	147.96		
9	127.8	7.21	C-4a, C-6, C-10a
10	127.4	7.81	C-5, C-11, C-14a
10a	130.9		
11	104.5	7.45 (s)	C-10, C-13, C-14a
12	148.0		
13	149.2		
14	98.9	6.98	C-6, C-12, C-10a
14a	128.9		
C-1 OMe	60.6	3.65 (s)	C-1
C-2-OMe	55.9	3.79(s)	C-2
O-CH_2-O	101.9	6.15(s)	C-12, C-13

The NMR assignments and HMBC correlations of 8-oxochelerythrine (**8**) isolated from the root bark of *Zanthoxylum paracanthum* observed at 500 MHz dissolved in $CDCl_3$.

1.3. NMR and MS Spectra of the isolated compounds 1-8

1.3.1. Myristic acid (1)

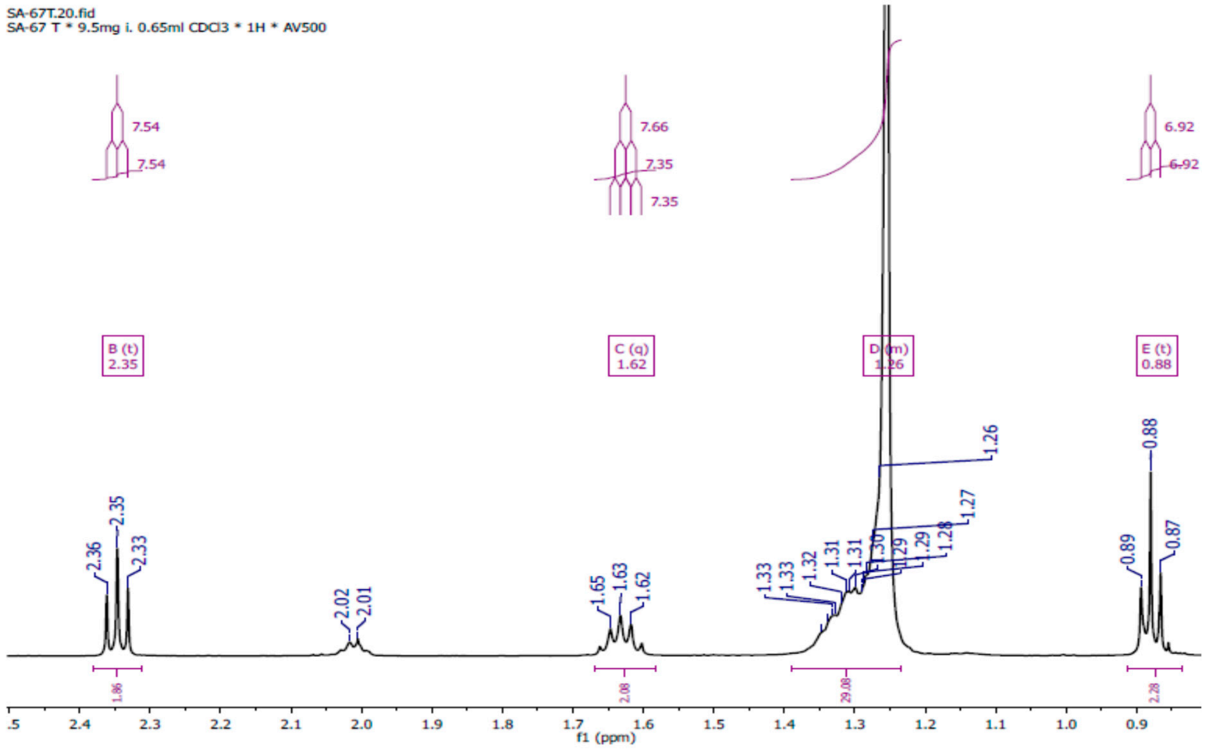

Figure S1. The ^{1}H NMR spectrum of myristic acid (**1**) observed at 500 MHz for $CDCl_3$ solution at 25 °C. Assignment is given in Table S1.

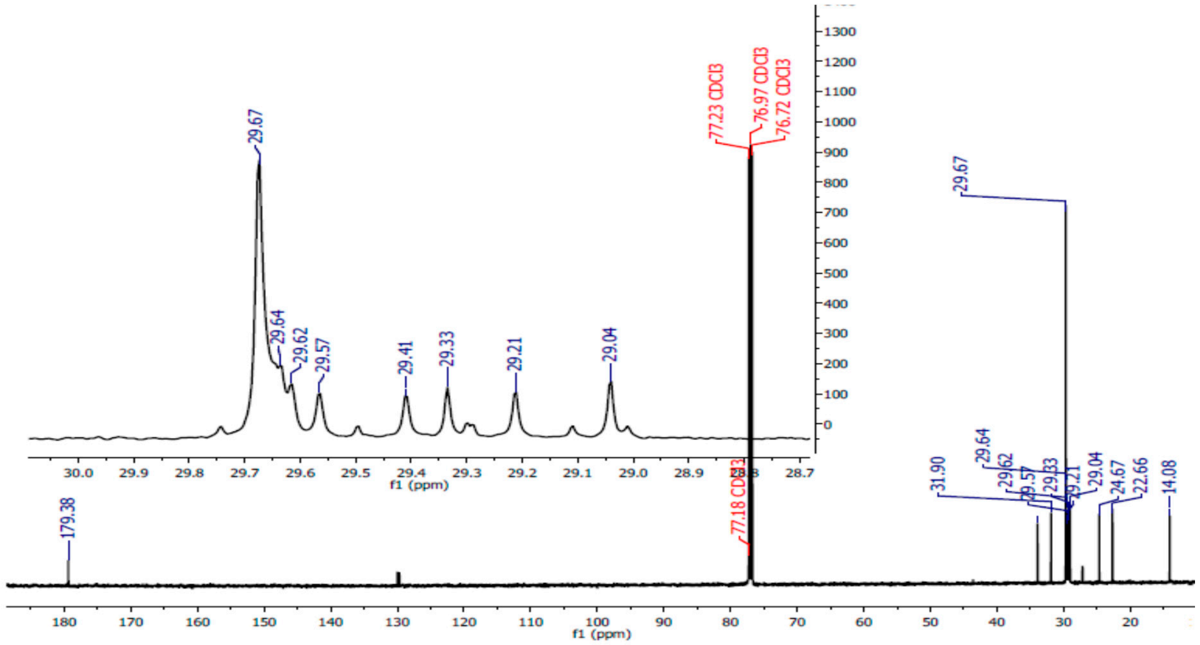

Figure S2. The ^{13}C NMR spectrum of myristic acid (**1**) observed at 125 MHz for $CDCl_3$ solution at 25 °C. Assignment is given in Table S1.

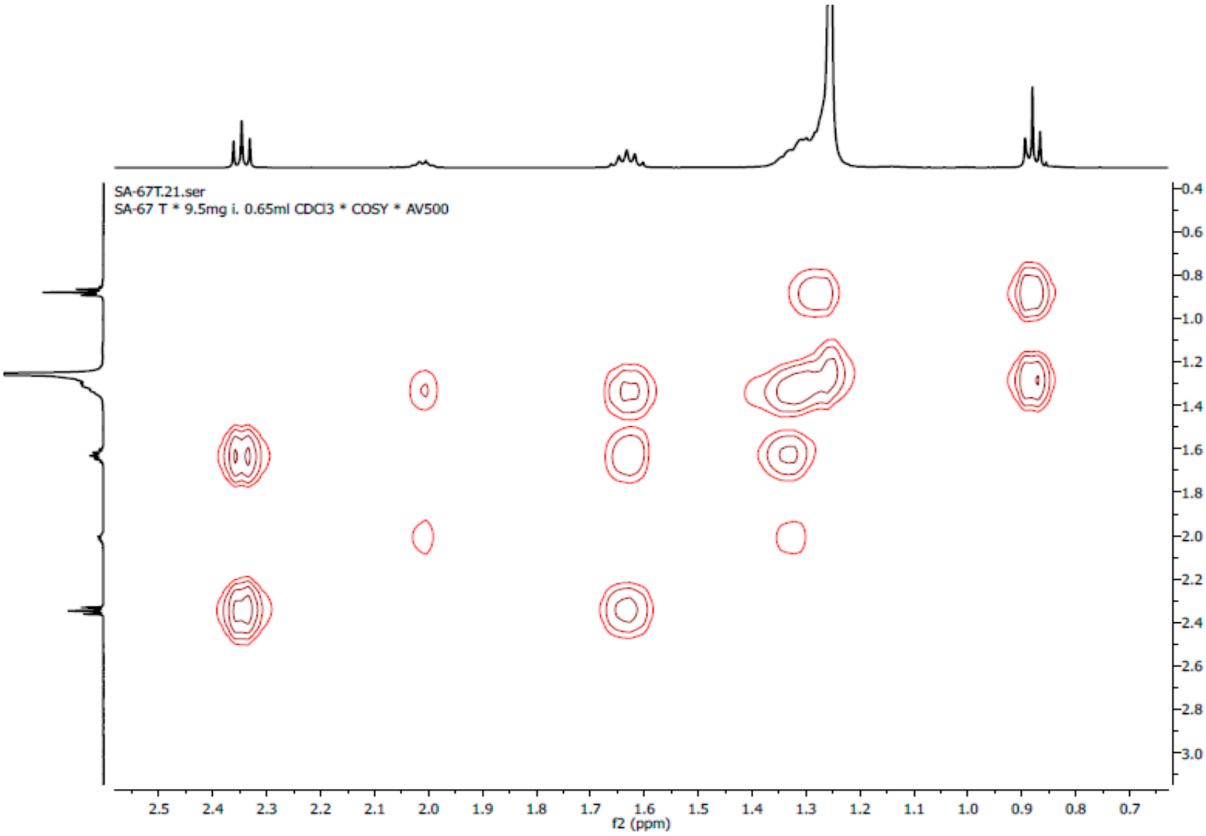

Figure S3. The ^{1}H-^{1}H COSY spectrum of myristic acid (**1**) observed at 500 MHz for $CDCl_3$ solution at 25 °C.

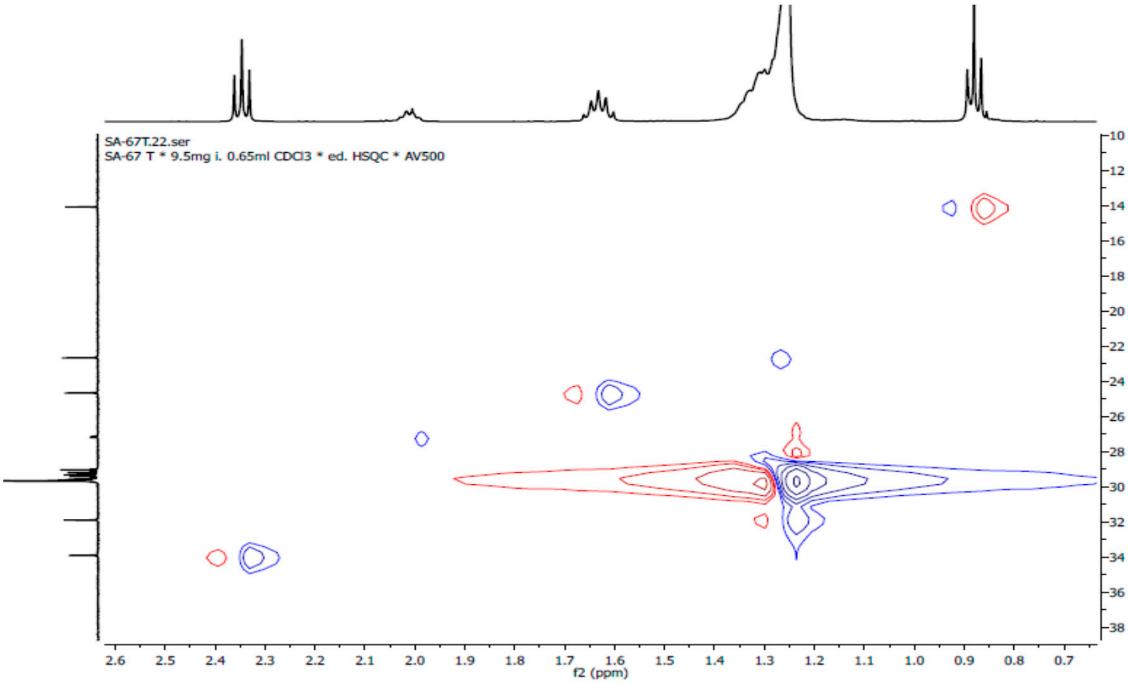

Figure S4. The ^{1}H-^{13}C HSQC NMR spectrum of myristic acid (**1**) observed at 500 and 125 MHz for $CDCl_3$ solution at 25 °C.

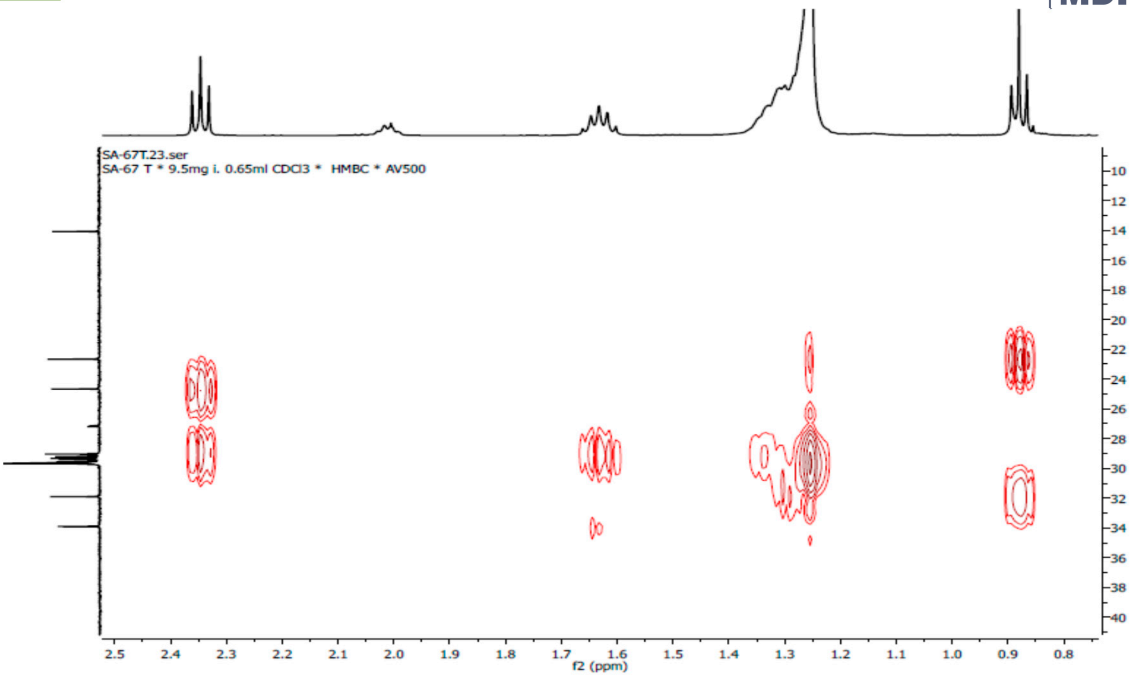

Figure S5. The ^{1}H-^{13}C HMBC NMR spectrum of myristic acid (**1**) observed at 500 and 125 MHz for $CDCl_3$ solution at 25 °C. Assignment is given in Table S1.

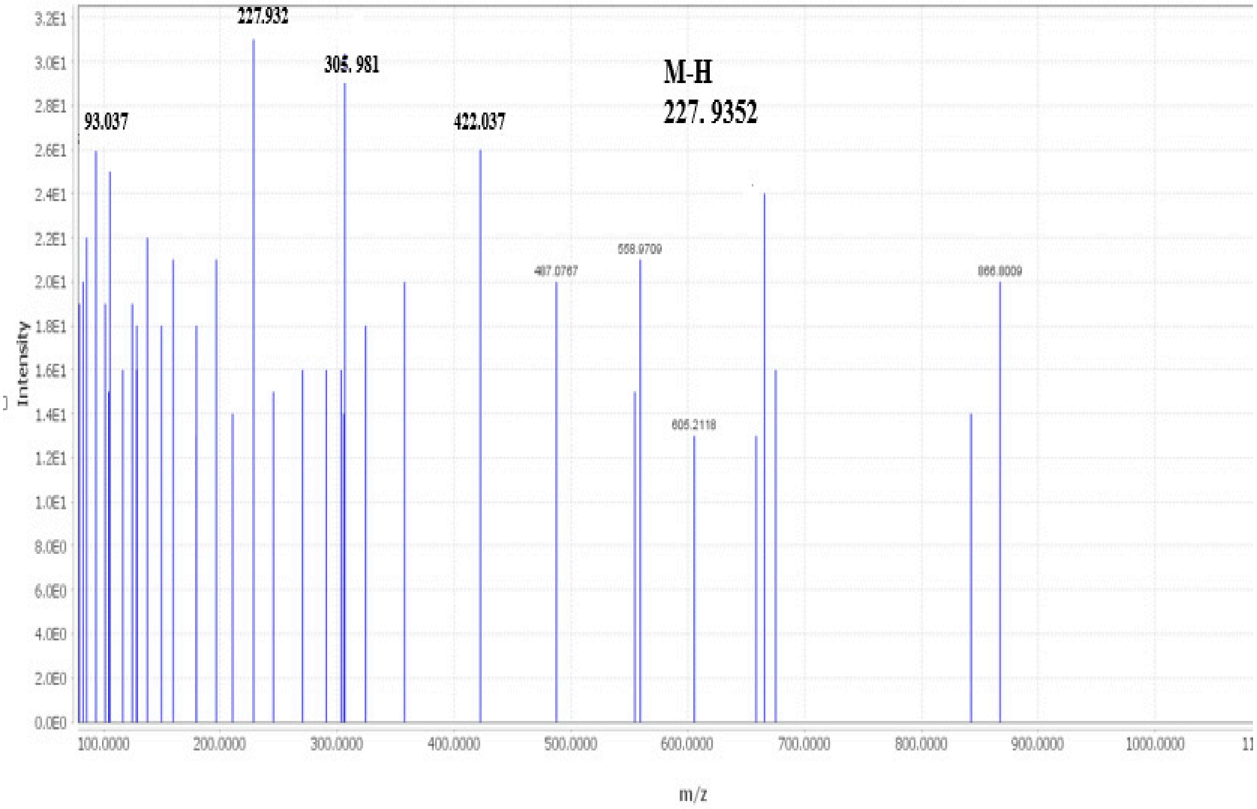

Figure S6. The ESIMS spectrum for myristic acid (**1**).

1.3.2. Stigmasterol (2)

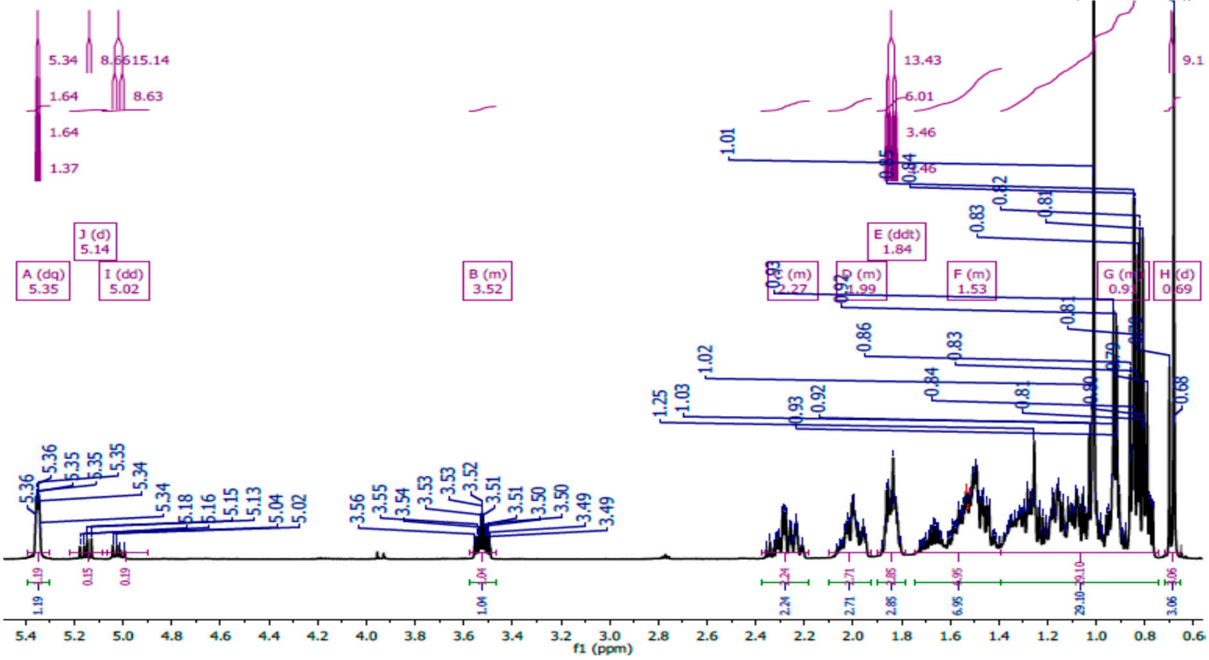

Figure S7. The ^{1}H NMR spectrum of stigmasterol (**2**) observed at 500 MHz for $CDCl_3$ solution at 25 °C. Assignment is given in Table S2.

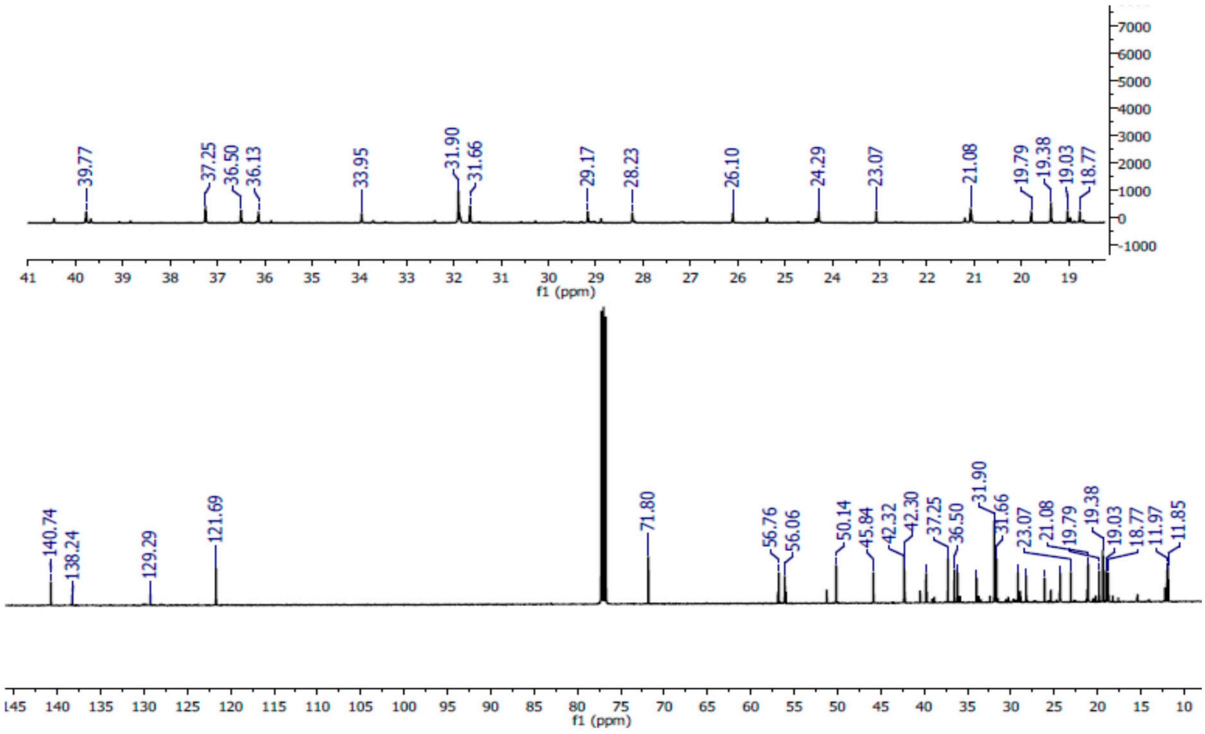

Figure S8. The ^{13}C NMR spectrum of stigmasterol (**2**) observed at 125 MHz for $CDCl_3$ solution at 25 °C. Assignment is given in Table S2.

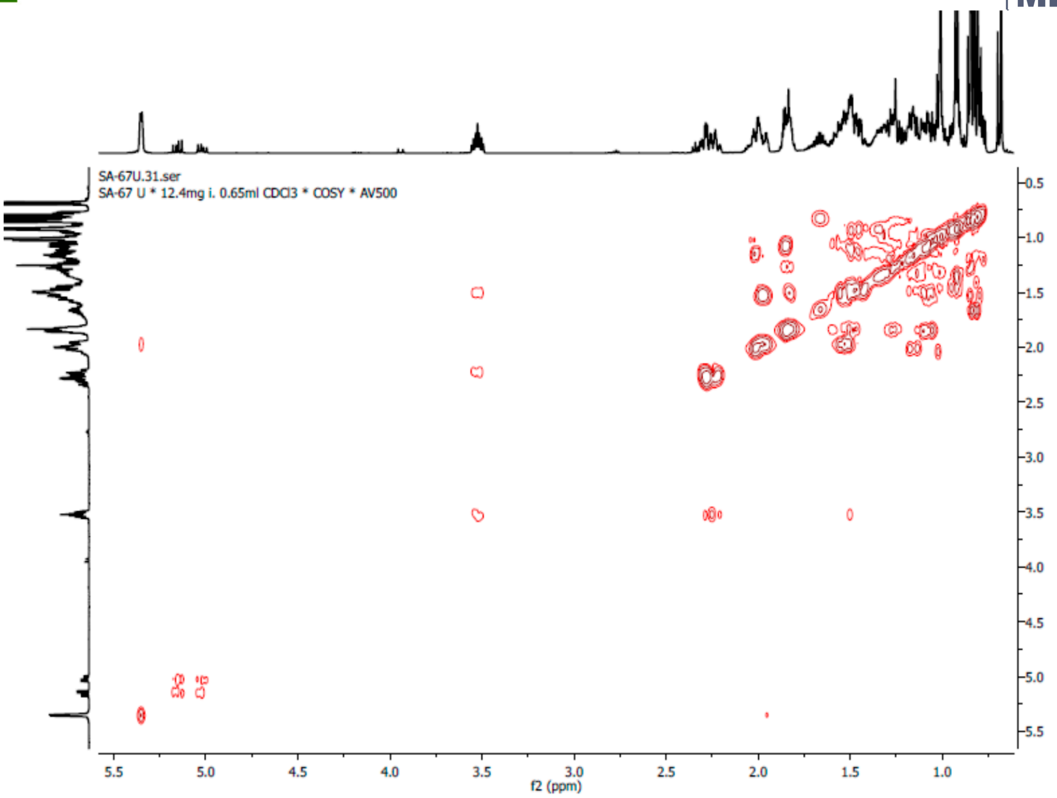

Figure S9. The ^{1}H-^{1}H COSY spectrum of stigmasterol (**2**) observed at 500 MHz for $CDCl_3$ solution at 25 °C.

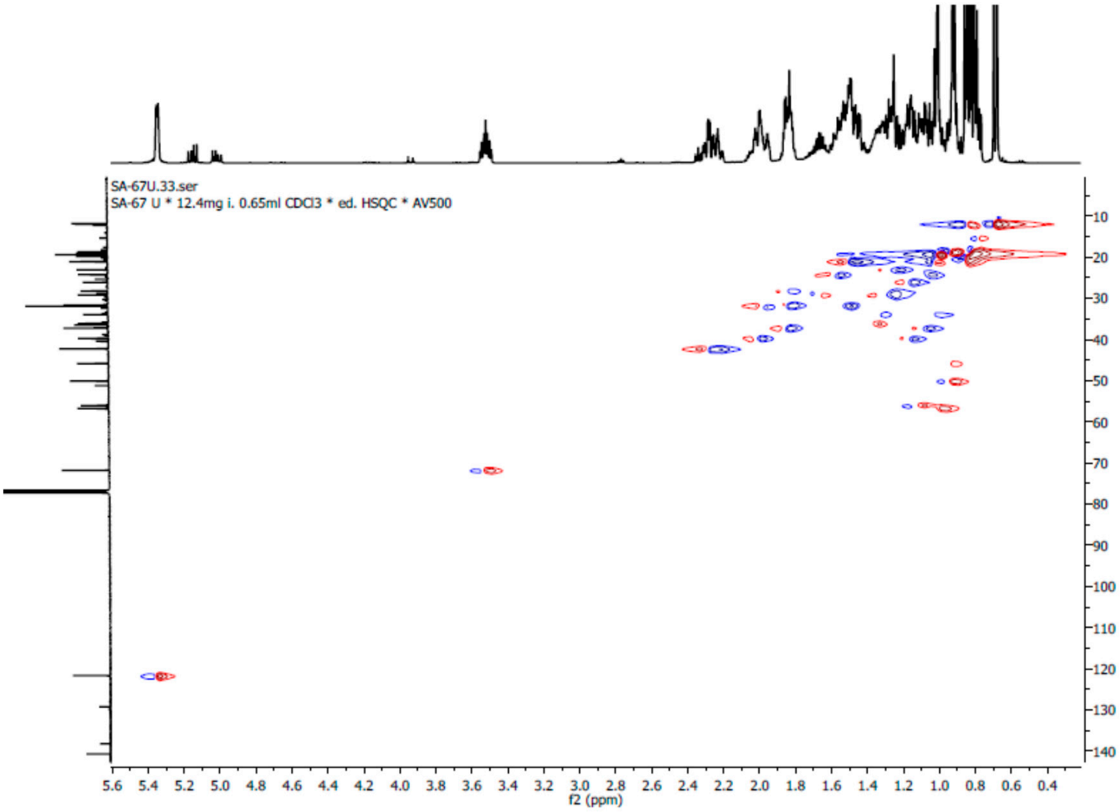

Figure S10. The ^{1}H-^{13}C HSQC NMR spectrum of stigmasterol (**2**) observed at 500 and 125 MHz for $CDCl_3$ solution at 25 °C.

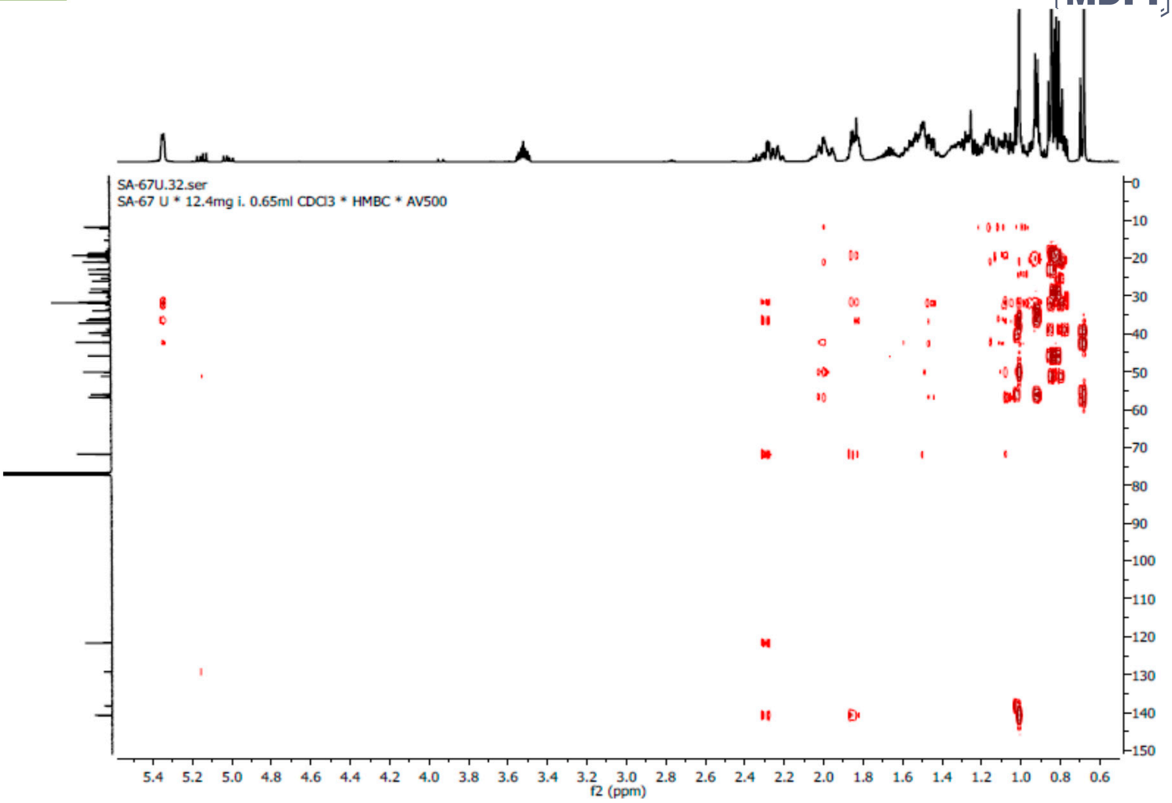

Figure S11. The ^{1}H-^{13}C HMBC NMR spectrum of stigmasterol (**2**) observed at 500 and 125 MHz for $CDCl_3$ solution at 25 °C. Assignment is given in Table S2.

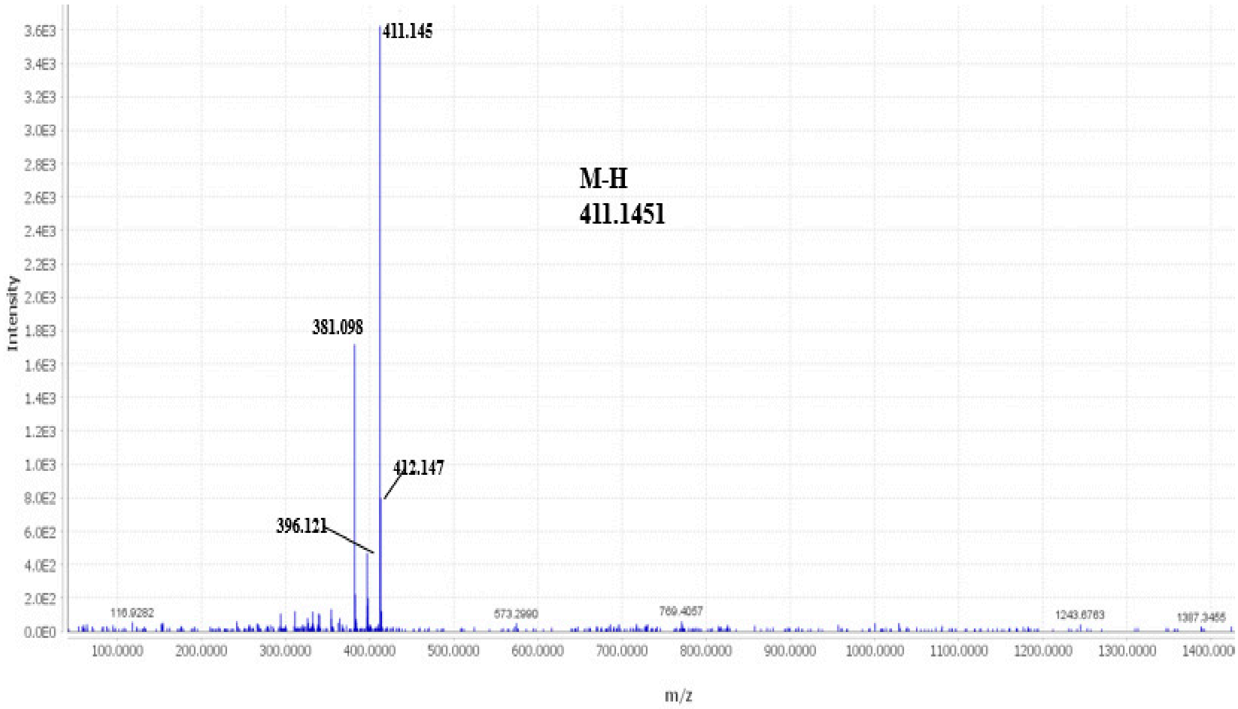

Figure S12. The ESIMS spectrum of stigmasterol (**2**).

1.3.3. Sesamin (3)

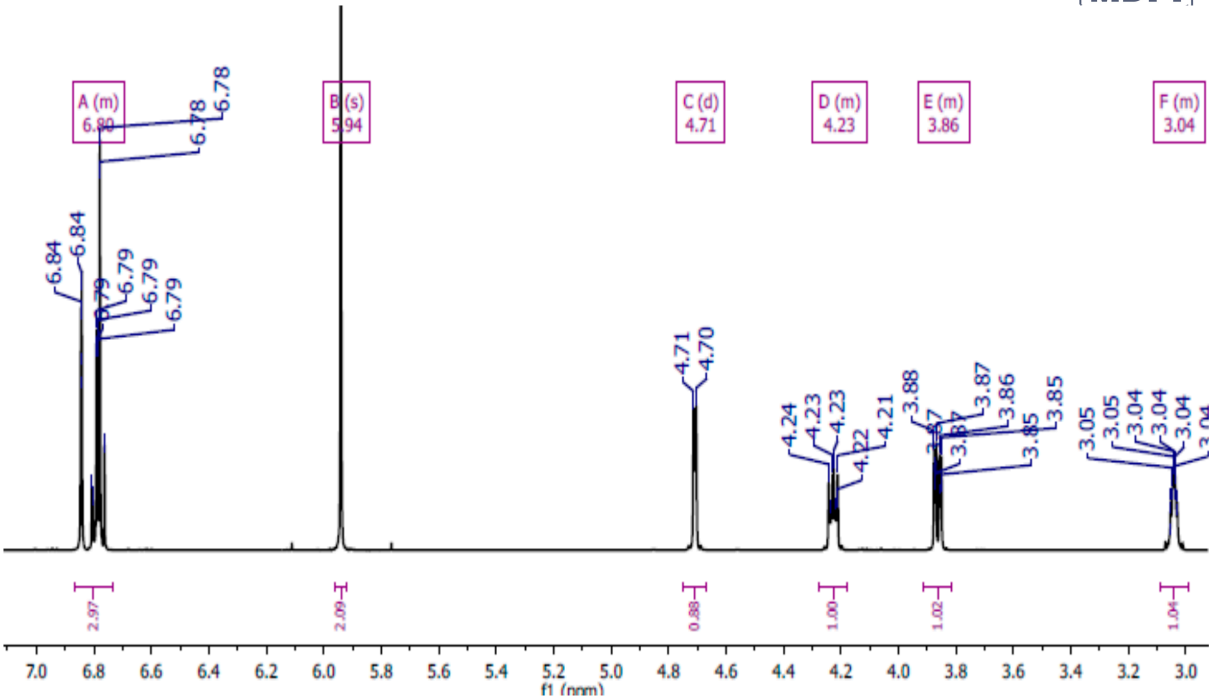

Figure S13. The ^{1}H NMR spectrum of sesamin (**3**) observed at 500 MHz for $CDCl_3$ solution at 25 °C. Assignment is given in Table S3.

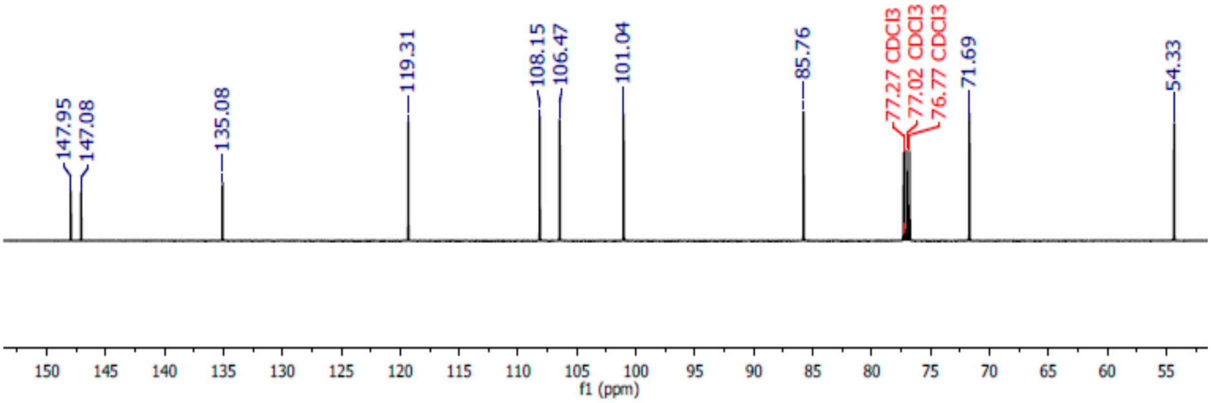

Figure S14. The ^{13}C NMR spectrum of sesamin (**3**) observed at 125 MHz for $CDCl_3$ solution at 25 °C. Assignment is given in Table 1. Assignment is given in Table S3.

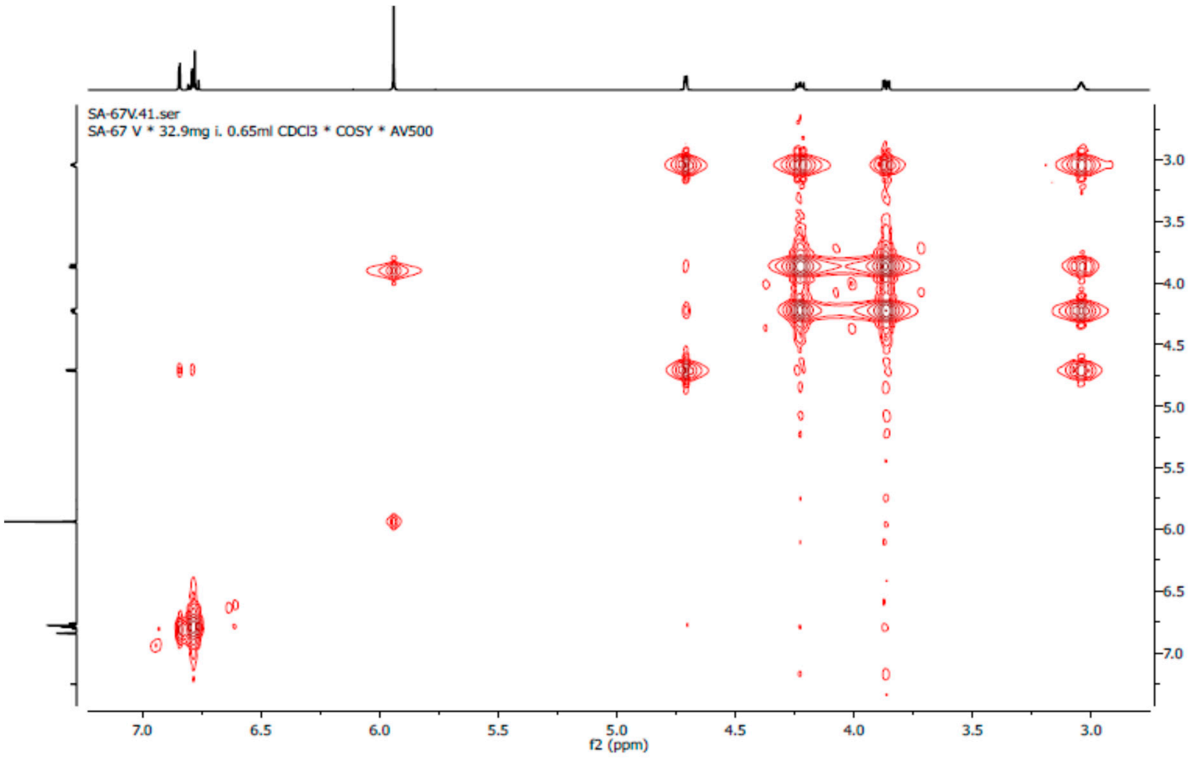

Figure S15. The ^{1}H-^{1}H COSY spectrum of sesamin (**3**) observed at 500 MHz for $CDCl_3$ solution at 25 °C.

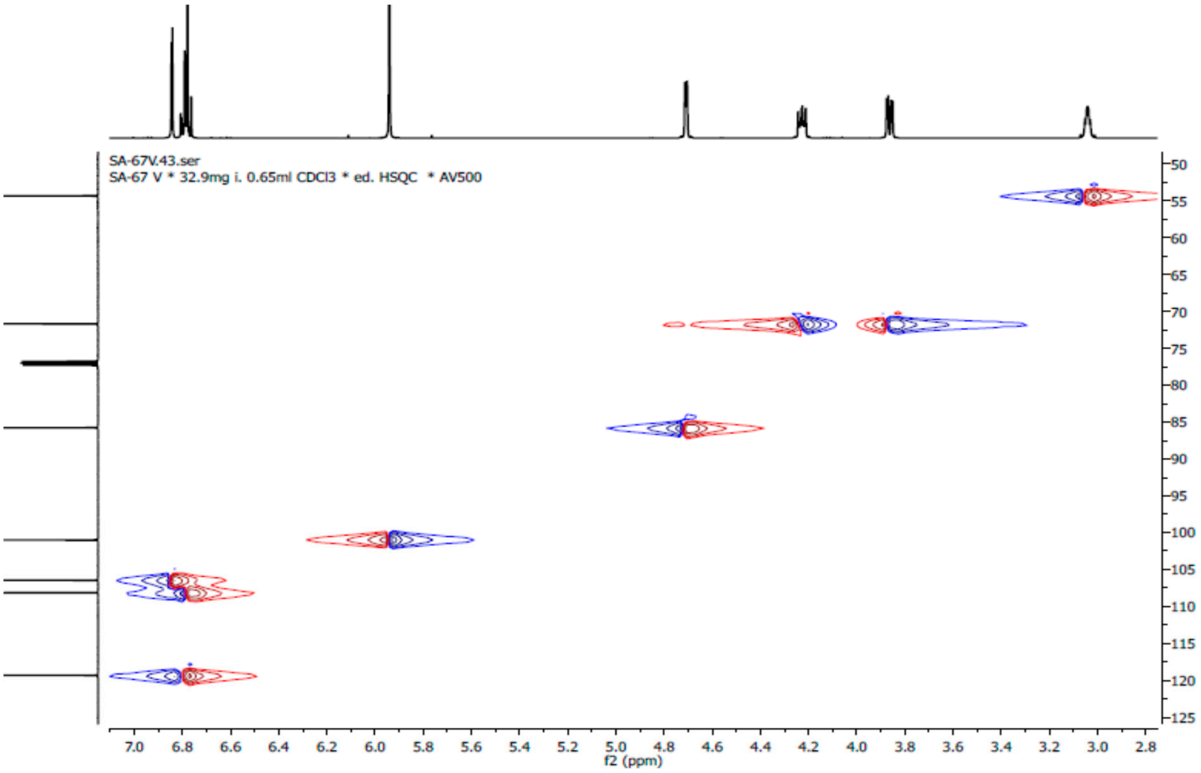

Figure S16. The ^{1}H-^{13}C HSQC NMR spectrum of sesamin (**3**) observed at 500 and 125 MHz for $CDCl_3$ solution at 25 °C.

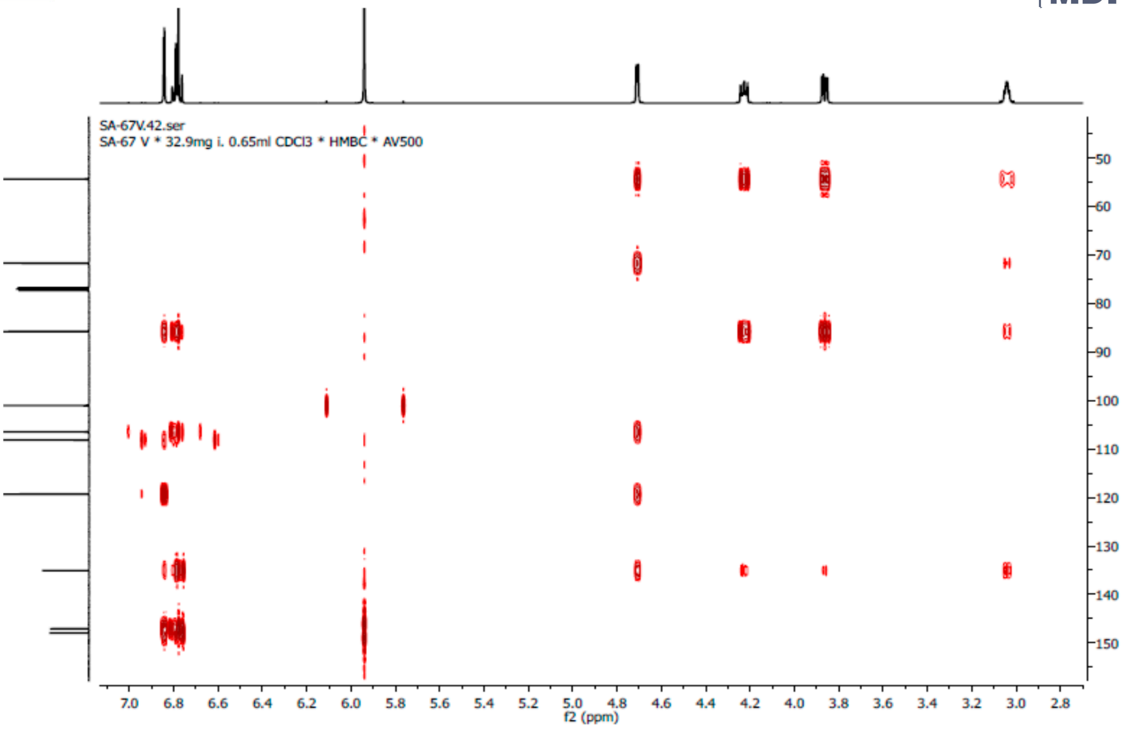

Figure S17. The ^{1}H-^{13}C HMBC NMR spectrum of sesamin (**3**) observed at 500 and 125 MHz for $CDCl_3$ solution at 25 °C. Assignment is given in Table S3.

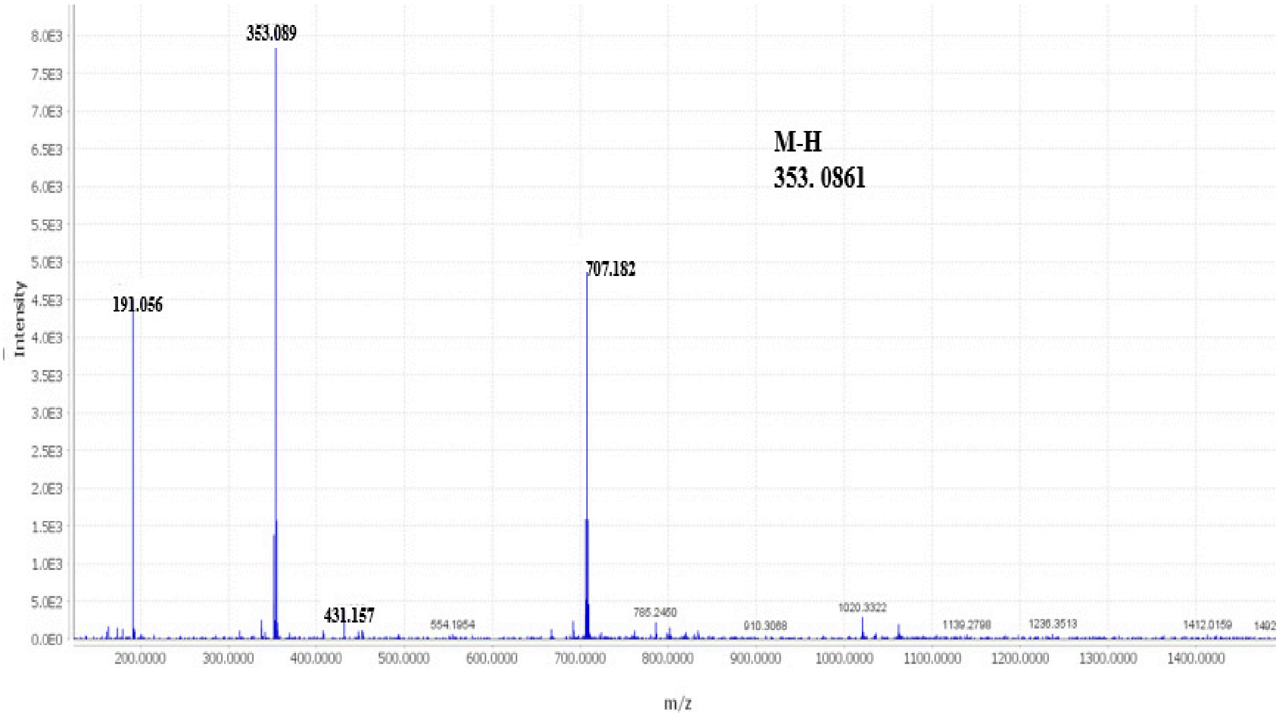

Figure S18. The ESIMS spectrum of sesamin (**3**).

1.3.4. 8-Acetonyldihydrochelerythrine (4)

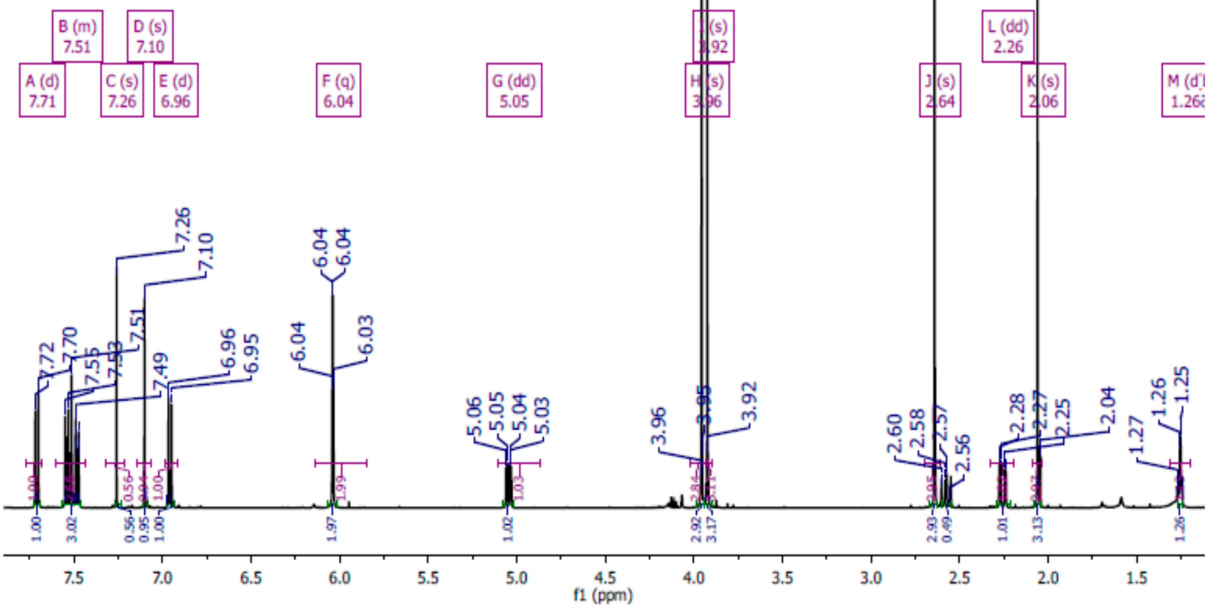

Figure S19. The ^{1}H NMR spectrum of 8-acetonyldihydrochelerythrine (**4**) observed at 500 MHz for $CDCl_3$ solution at 25 °C. Assignment is given in Table S4.

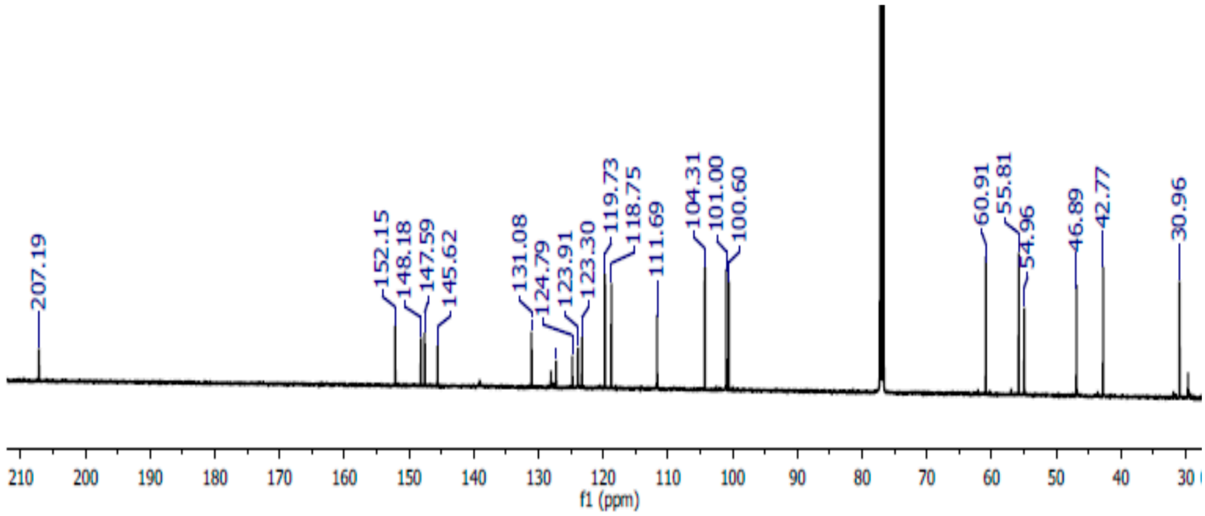

Figure S20. The ^{13}C NMR spectrum of 8-acetonyldihydrochelerythrine (**4**) observed at 125 MHz for $CDCl_3$ solution at 25 °C. Assignment is given in Table S4.

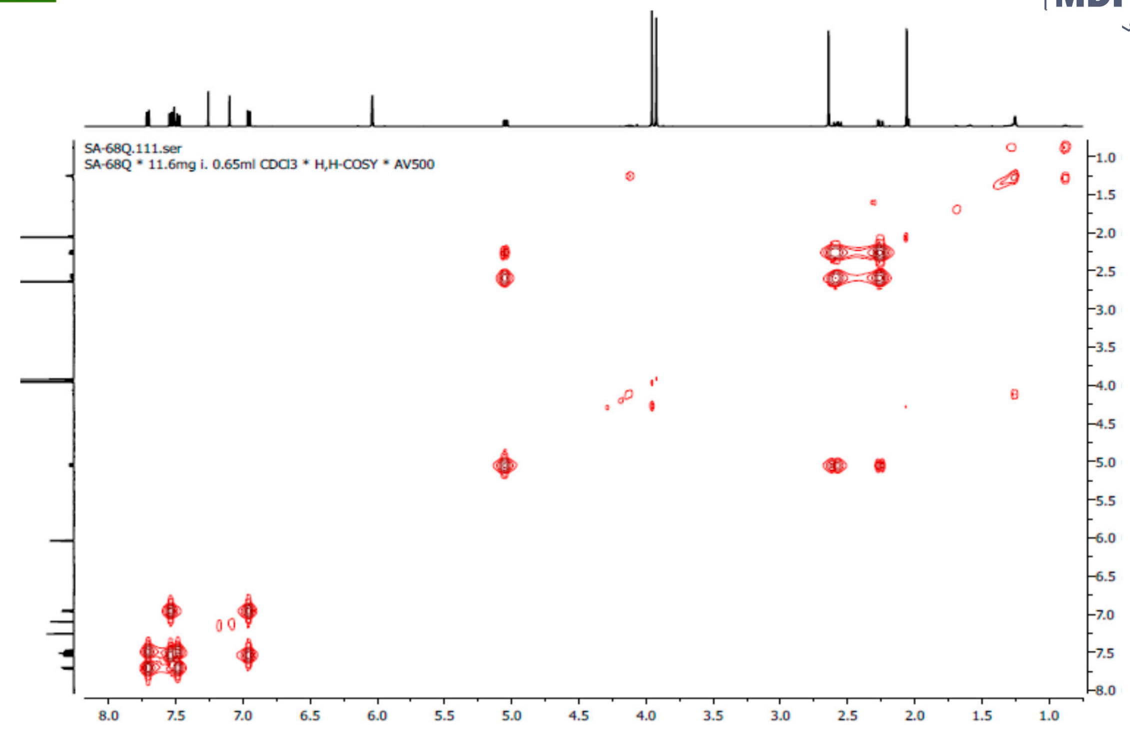

Figure S21. The ^{1}H-^{1}H COSY spectrum of 8-acetonyldihydrochelerythrine (**4**) observed at 500 MHz for $CDCl_3$ solution at 25 °C.

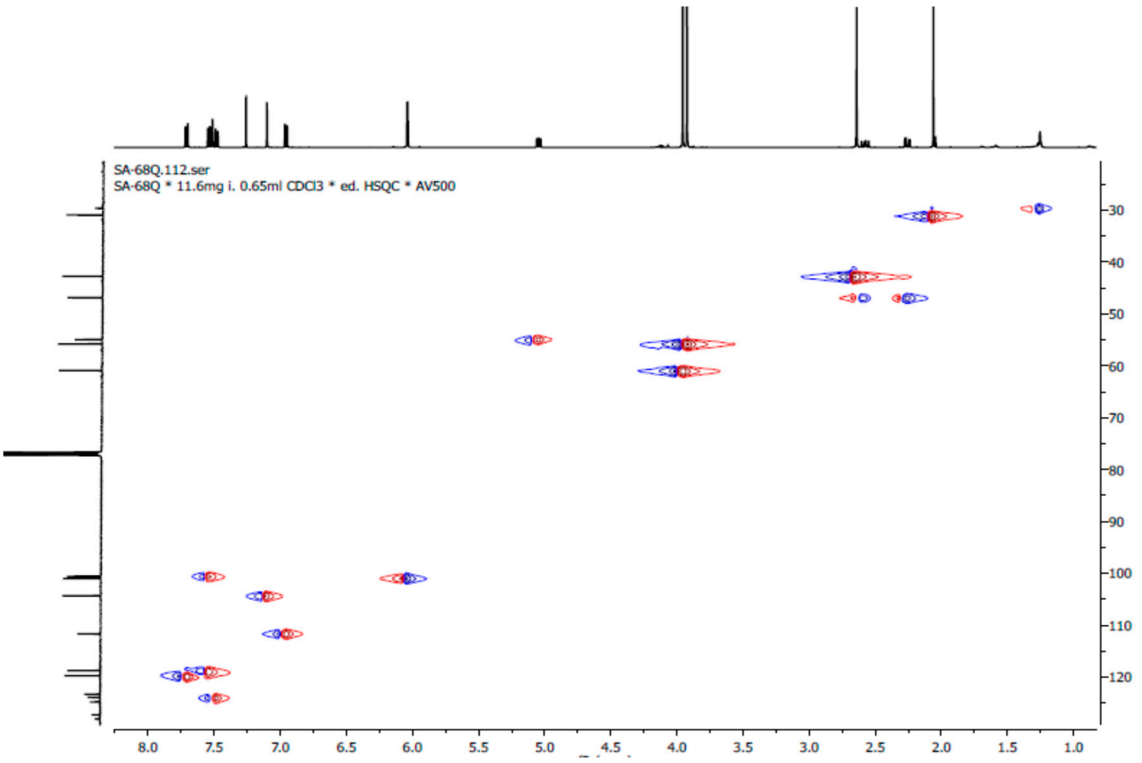

Figure S22. The ^{1}H-^{13}C HSQC NMR spectrum of 8-acetonyldihydrochelerythrine (**4**) observed at 500 and 125 MHz for $CDCl_3$ solution at 25 °C.

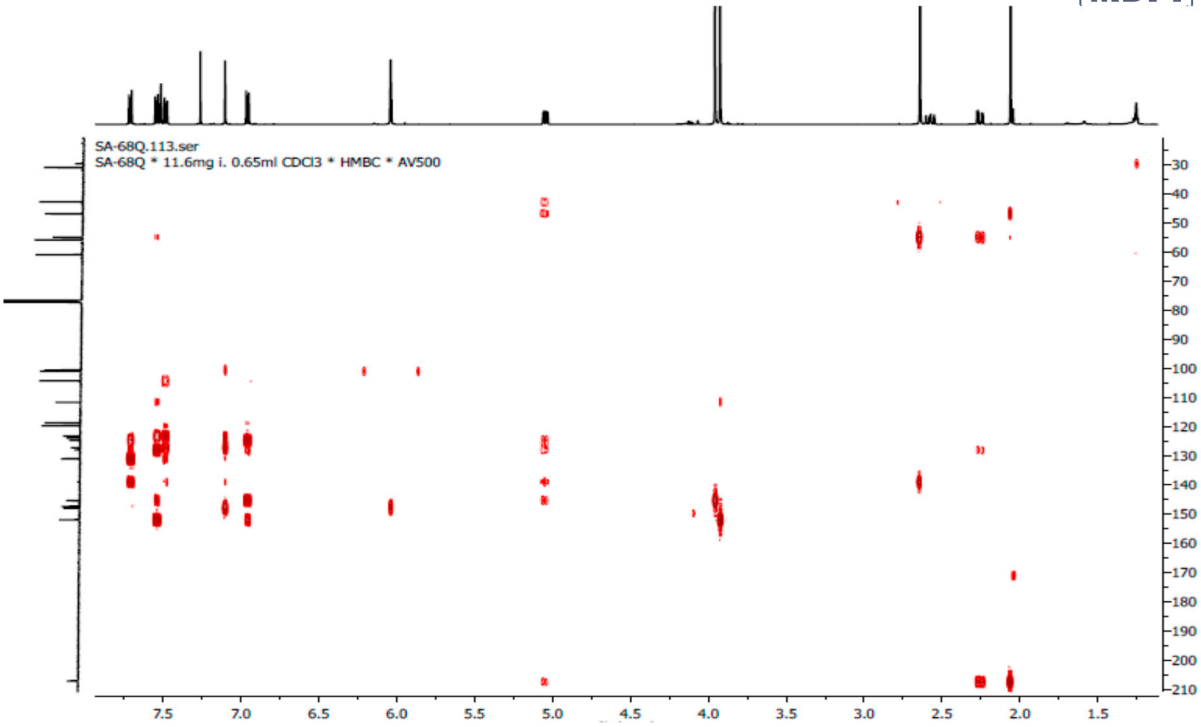

Figure S23. The ^{1}H-^{13}C HMBC NMR spectrum of 8-acetonyldihydrochelerythrine (**4**) observed at 500 and 125 MHz for $CDCl_3$ solution at 25 °C. Assignment is given in Table S4.

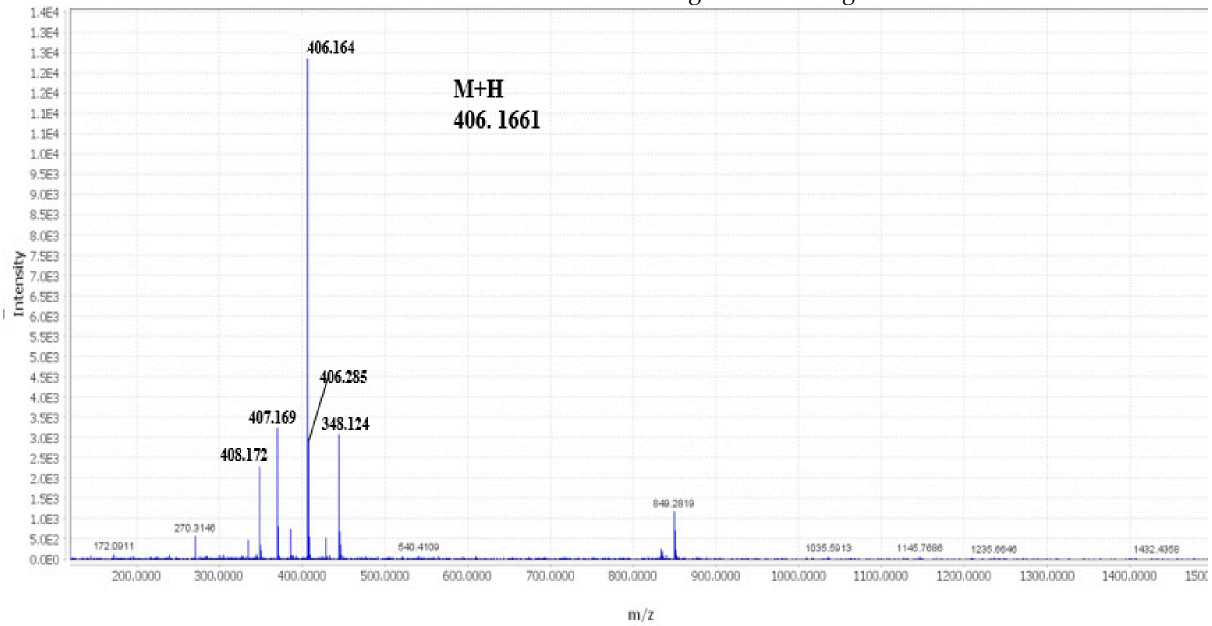

Figure S24. The ESIMS spectrum of 8-acetonyldihydrochelerythrine (**4**).

1.3.5. Arnottianamide (5)

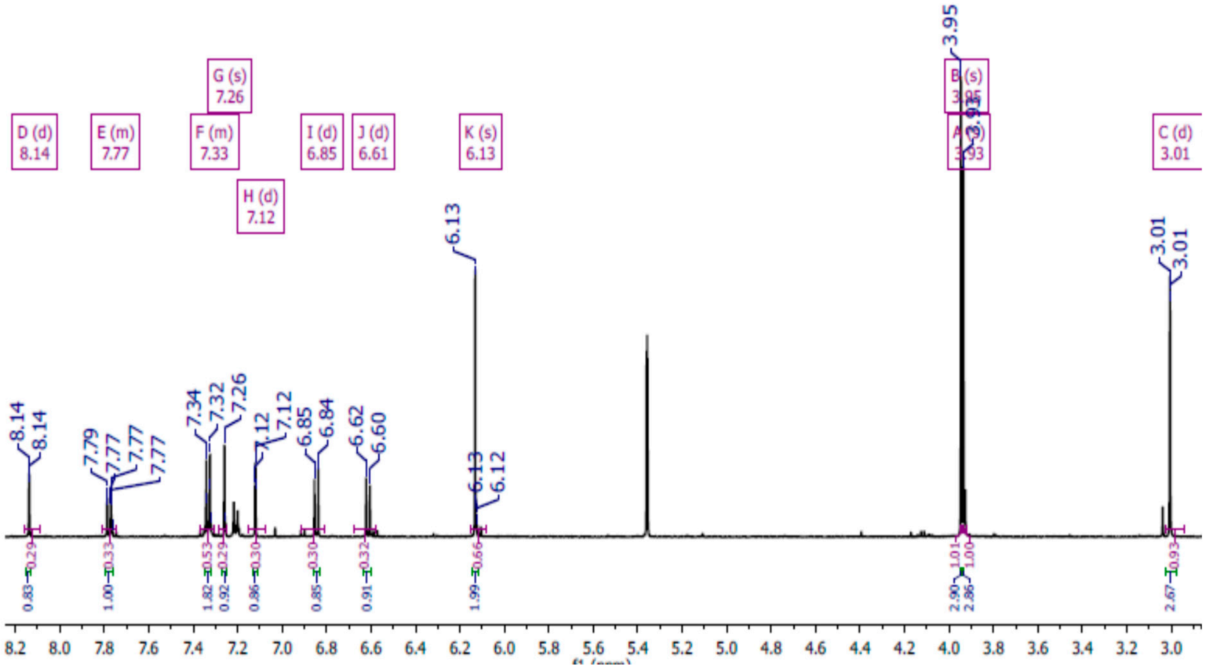

Figure S25. The ^{1}H NMR spectrum of arnottianamide (**5**) observed at 500 MHz for CD_2Cl_2 solution at 25 °C. Assignment is given in Table S5.

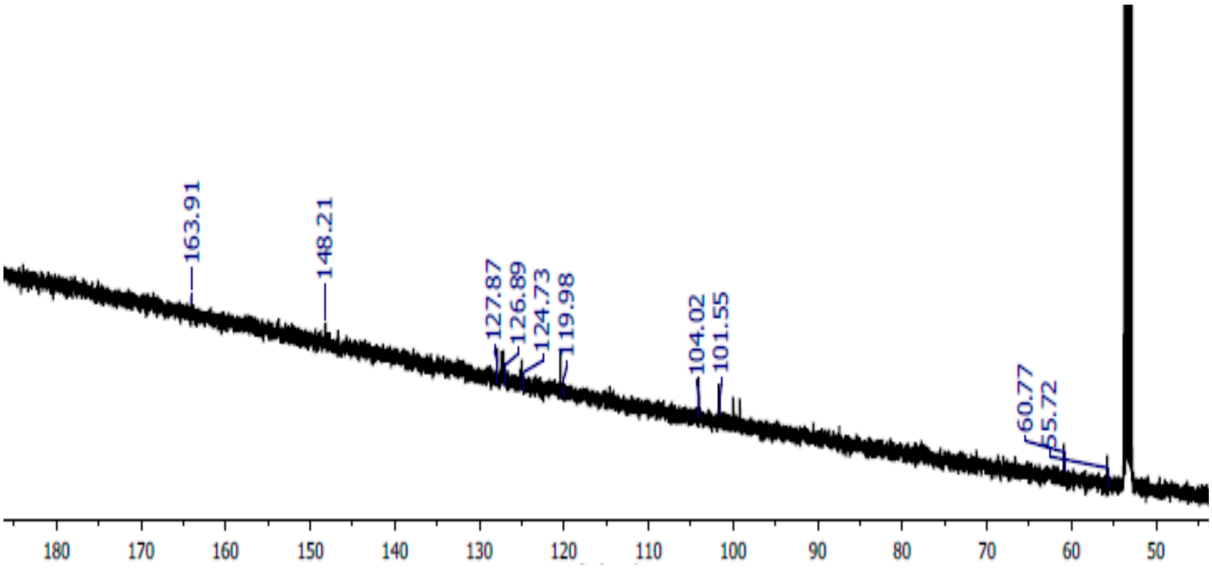

Figure S26. The ^{13}C NMR spectrum of arnottianamide (**5**) observed at 125 MHz for CD_2Cl_2 solution at 25 °C. Assignment is given in Table S5.

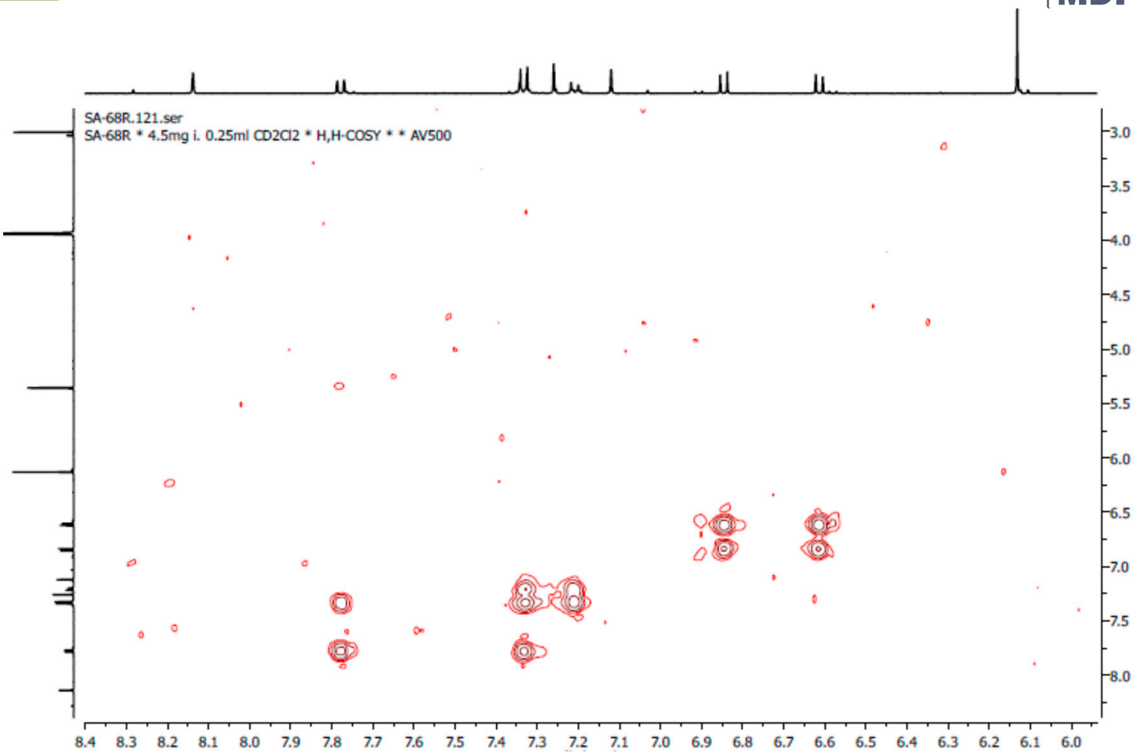

Figure S27. The ^{1}H-^{1}H COSY spectrum of arnottianamide (**5**) observed at 500 MHz for CD_2Cl_2 solution at 25 °C.

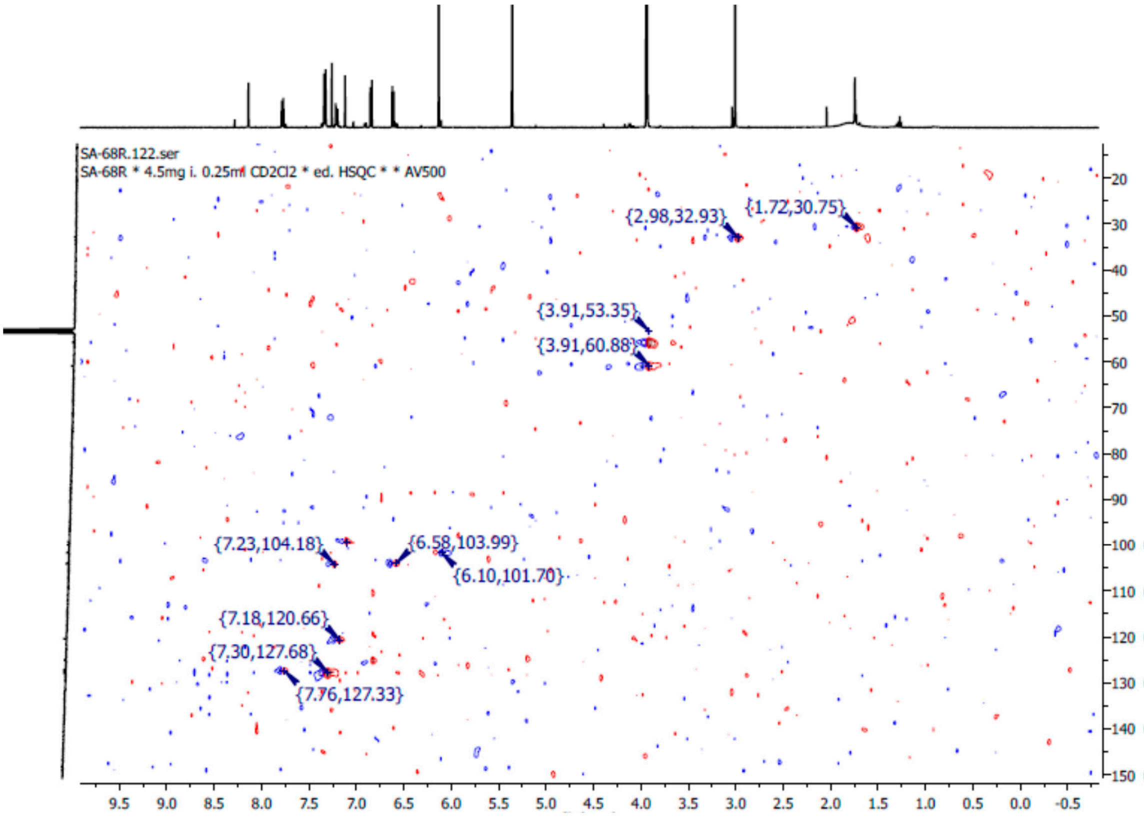

Figure S28. The ^{1}H-^{13}C HSQC NMR spectrum of arnottianamide (**5**) observed at 500 and 125 MHz for CD_2Cl_2 solution at 25 °C.

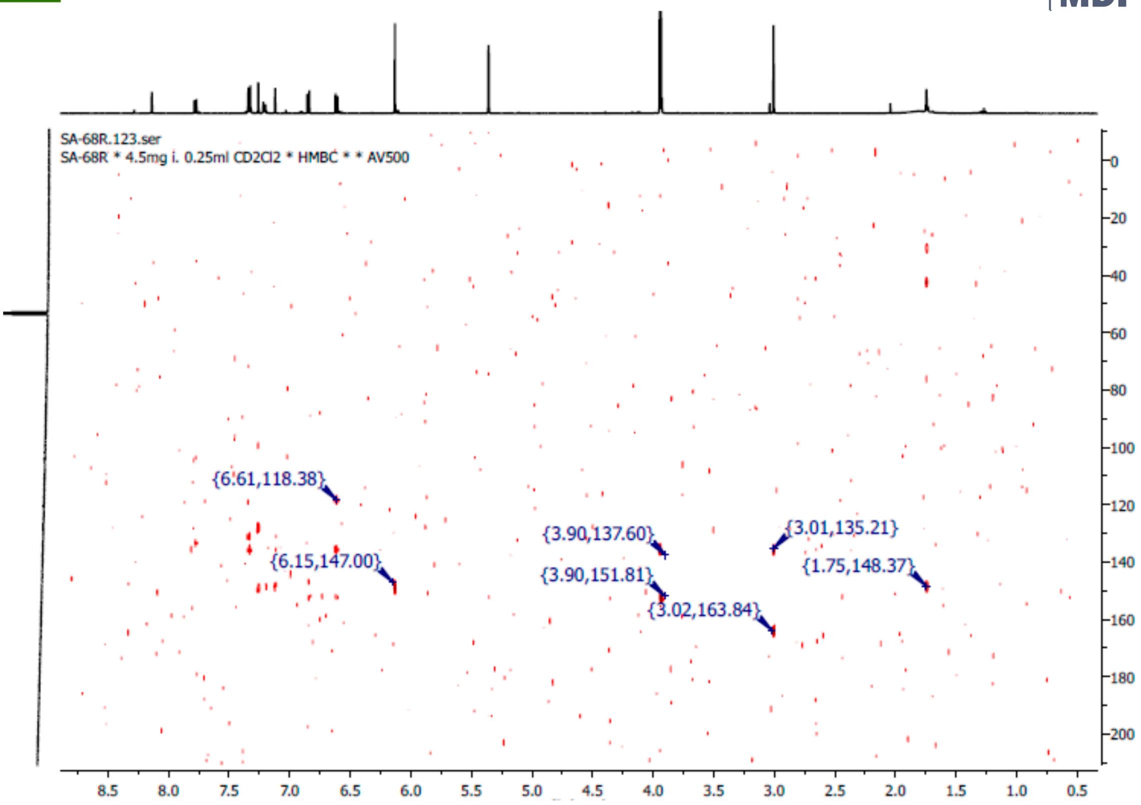

Figure S29. The ^1H-^{13}C HMBC NMR spectrum of arnottianamide (**5**) observed at 500 and 125 MHz for CD_2Cl_2 solution at 25 °C. Assignment is given in Table S5.

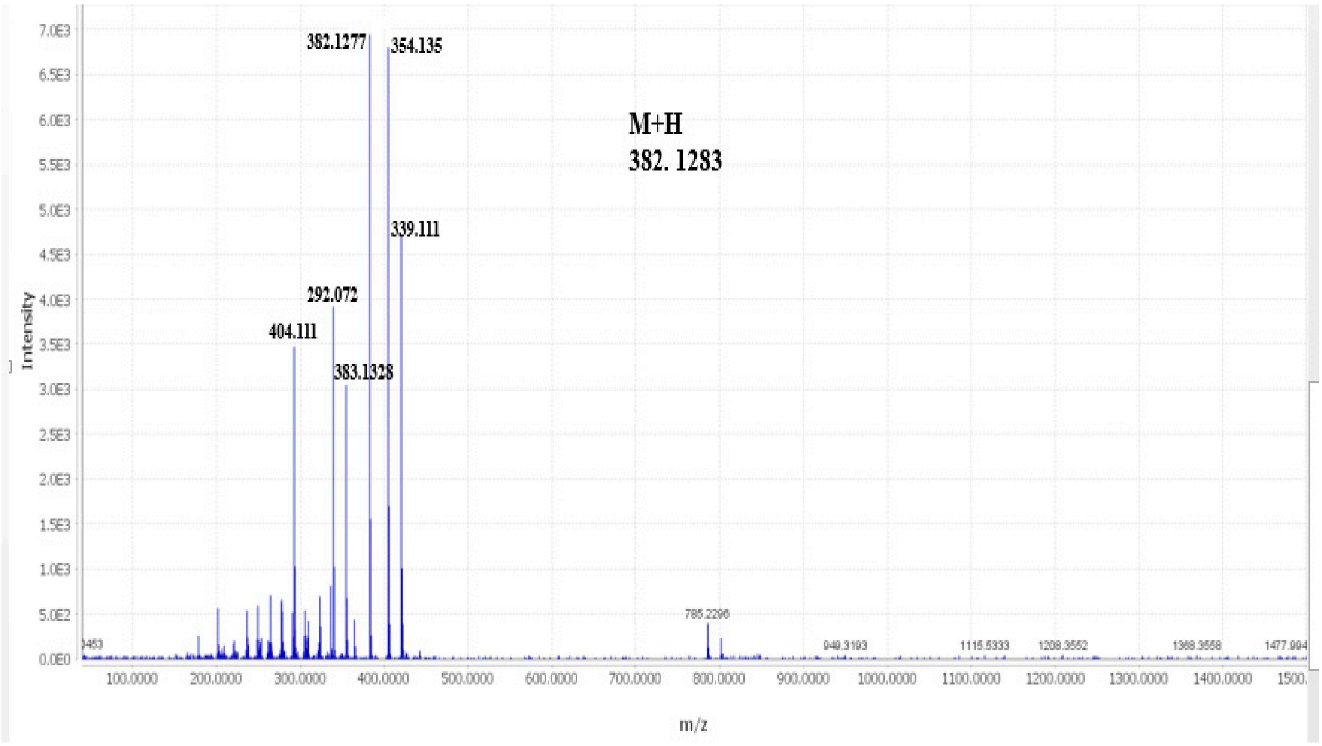

Figure S30. The ESIMS spectrum for arnottianamide (**5**).

1.3.6. 10-Methoxycanthn-6- one (6)

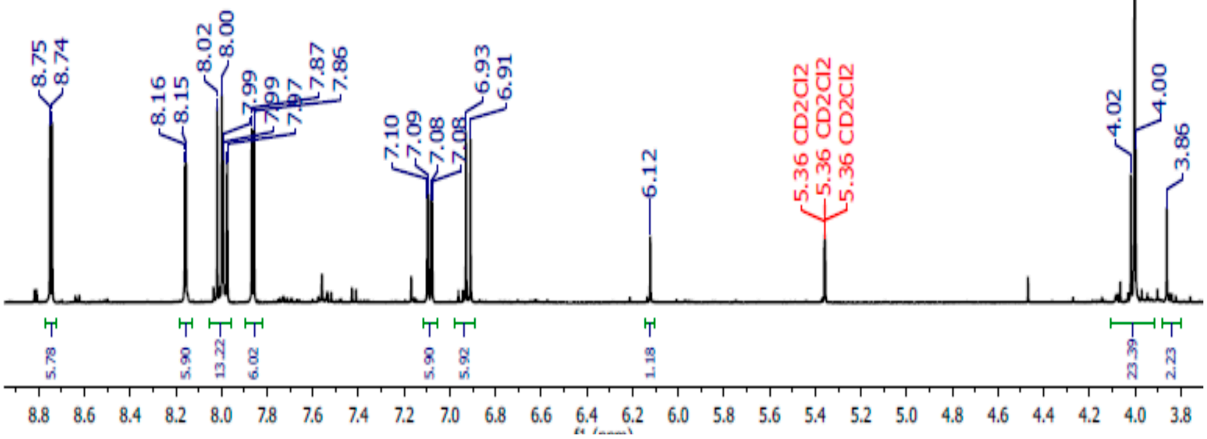

Figure S31. The ^{1}H NMR spectrum of 10-methoxycanthn-6- one (**6**) observed at 500 MHz for CD_2Cl_2 solution at 25 °C. Assignment is given in Table S6.

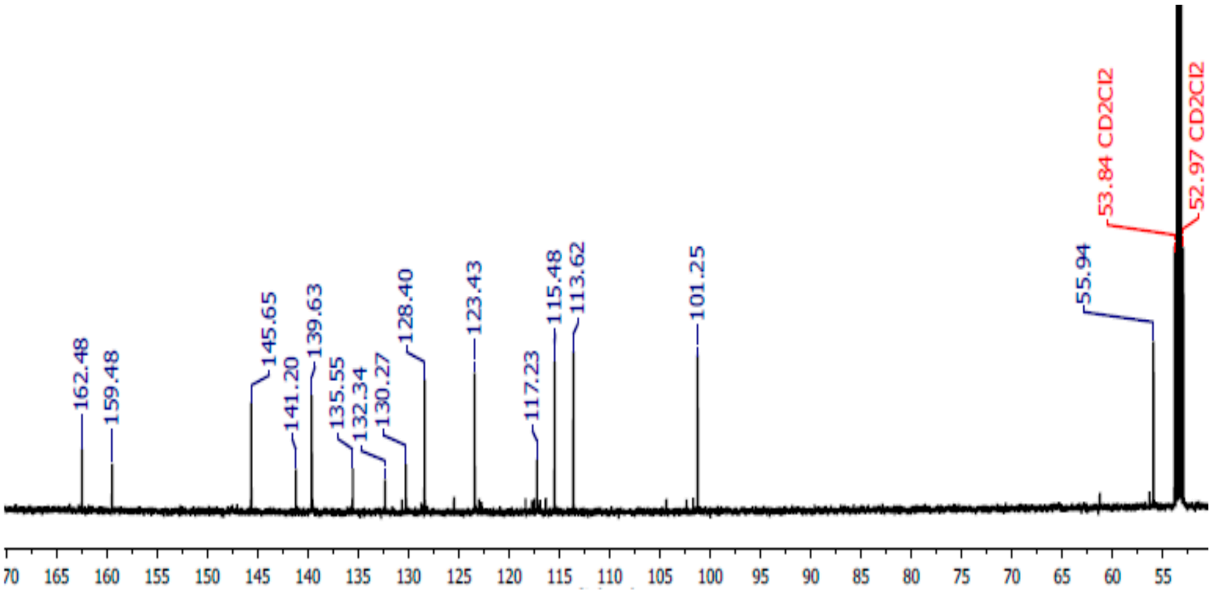

Figure S32. The ^{13}C NMR spectrum of 10-methoxycanthn-6- one (**6**) observed at 125 MHz for CD_2Cl_2 solution at 25 °C. Assignment is given in Table S6.

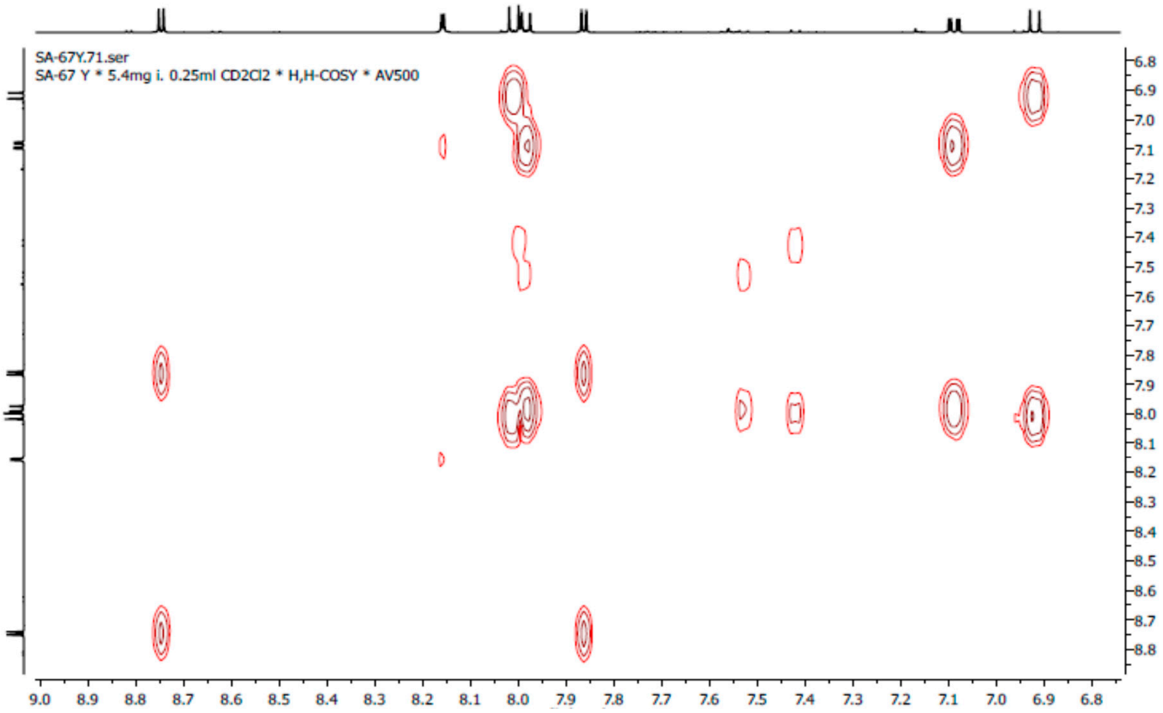

Figure S33. The ^{1}H-^{1}H COSY spectrum of 10-methoxycanthn-6- one (**6**) observed at 500 MHz for CD_2Cl_2 solution at 25 °C.

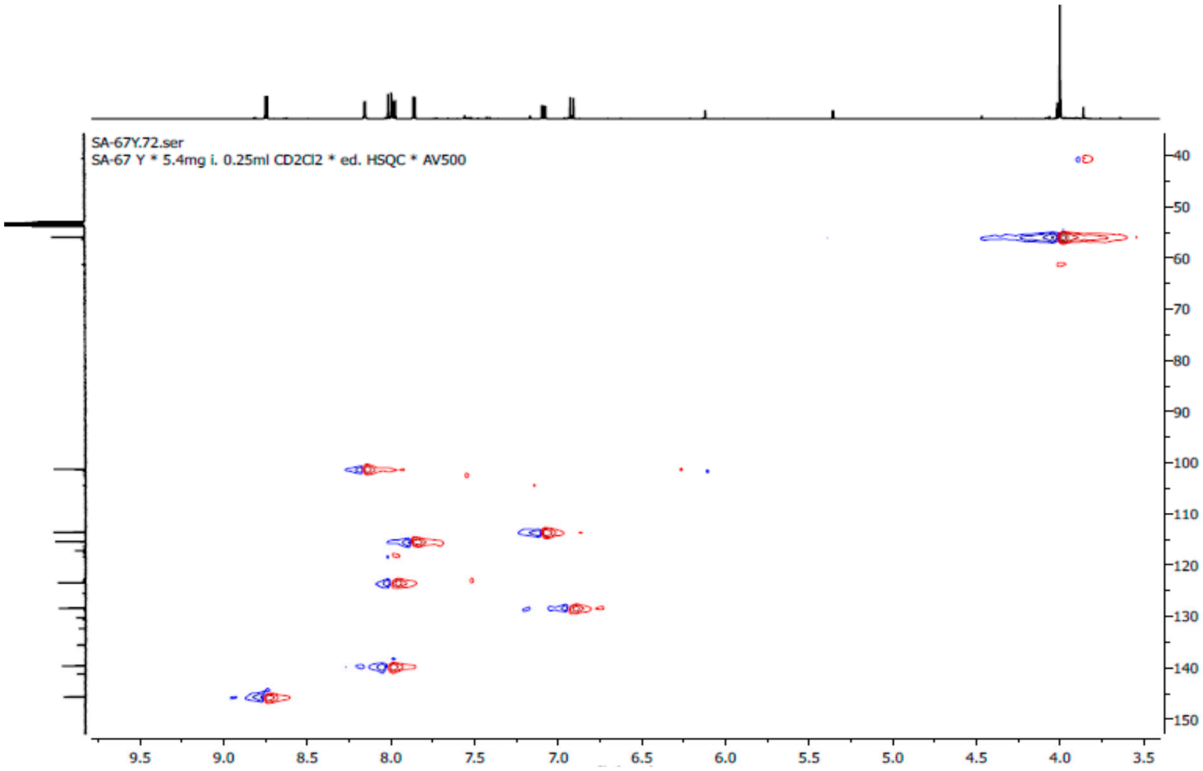

Figure S34. The ^{1}H-^{13}C HSQC NMR spectrum of 10-methoxycanthn-6- one (**6**) observed at 500 and 125 MHz for CD_2Cl_2 solution at 25 °C.

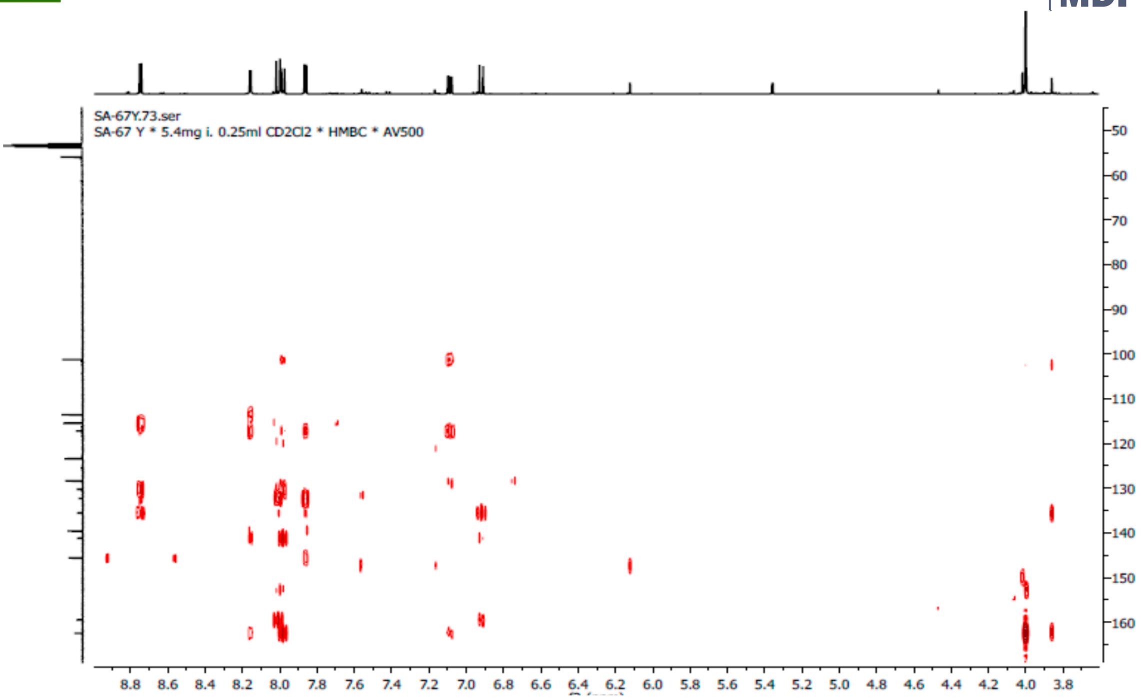

Figure S35. The ^{1}H-^{13}C HMBC NMR spectrum of 10-methoxycanthn-6- one (**6**) observed at 500 and 125 MHz for CD_2Cl_2 solution at 25 °C. Assignment is given in Table S6.

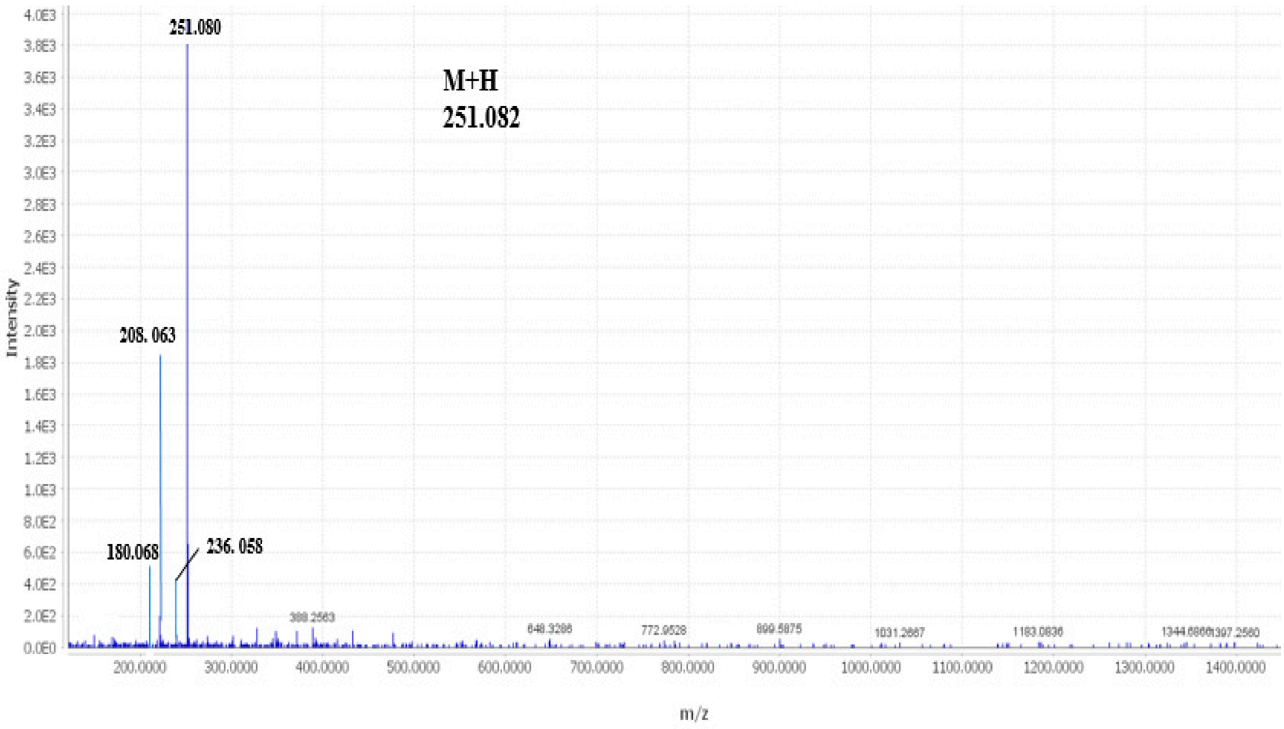

Figure S36. The ESIMS spectrum for 10-methoxycanthin- 6- one (**6**).

1.3.7. Canthin-6-one (7)

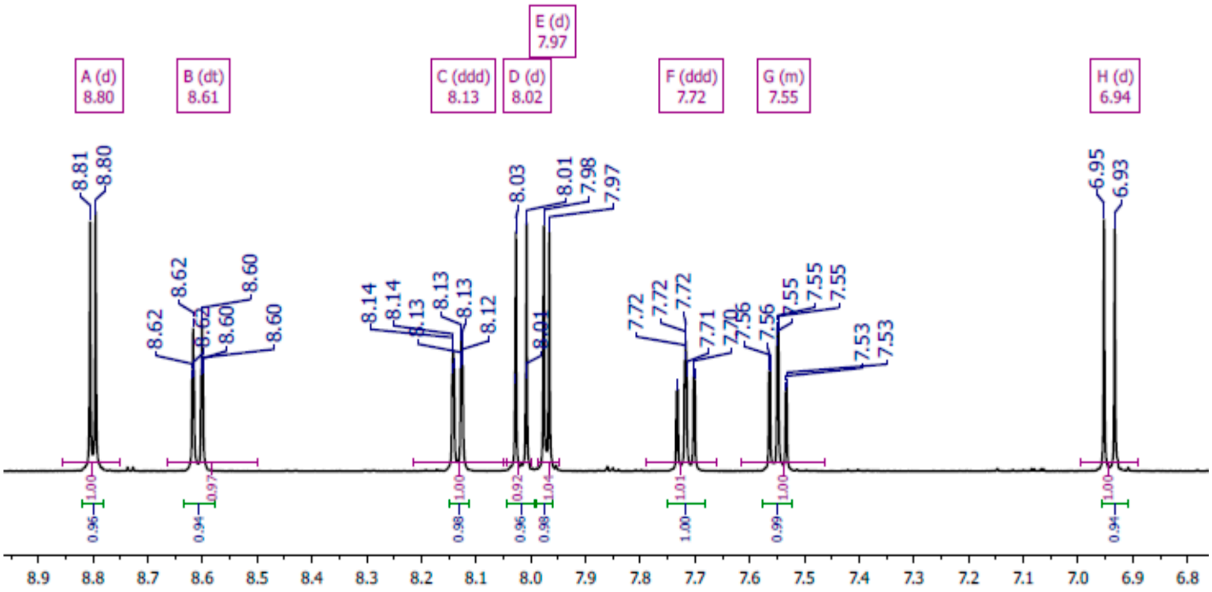

Figure S37. The ^{1}H NMR spectrum of canthin-6-one (**7**) observed at 500 MHz for CD_2Cl_2 solution at 25 °C. Assignment is given in Table S7.

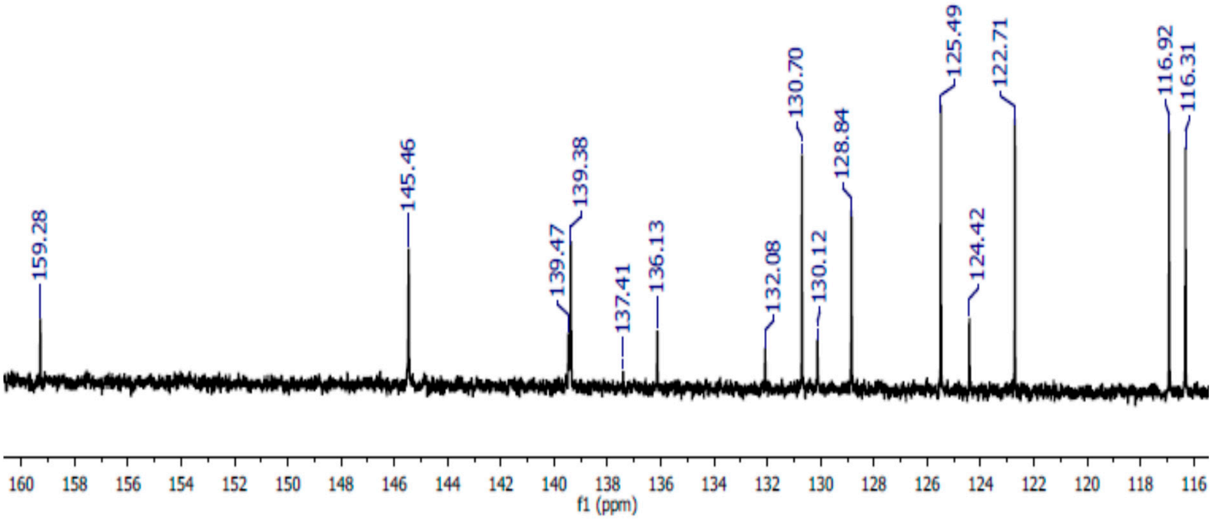

Figure S38. The ^{13}C NMR spectrum of canthin-6-one (**7**) observed at 125 MHz for CD_2Cl_2 solution at 25 °C. Assignment is given in Table S7.

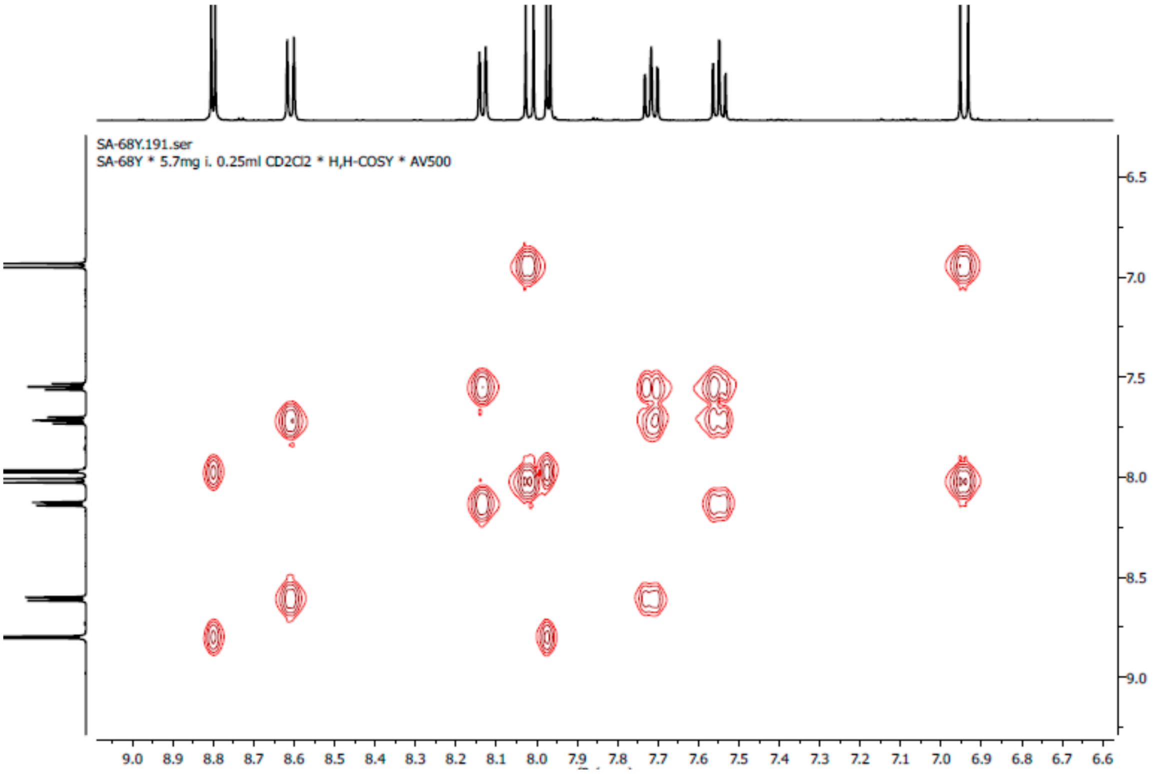

Figure S39. The ^{1}H-^{1}H COSY spectrum of canthin-6-one (**7**) observed at 500 MHz for CD_2Cl_2 solution at 25 °C.

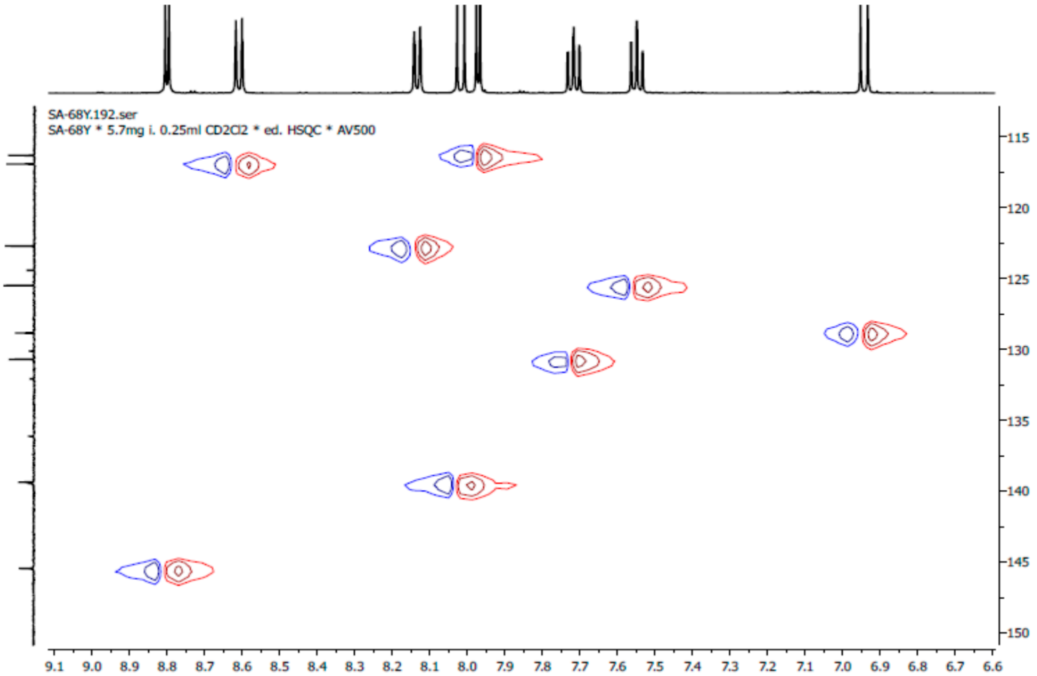

Figure S40. The ^{1}H-^{13}C HSQC NMR spectrum of canthin-6-one (**7**) observed at 500 and 125 MHz for CD_2Cl_2 solution at 25 °C.

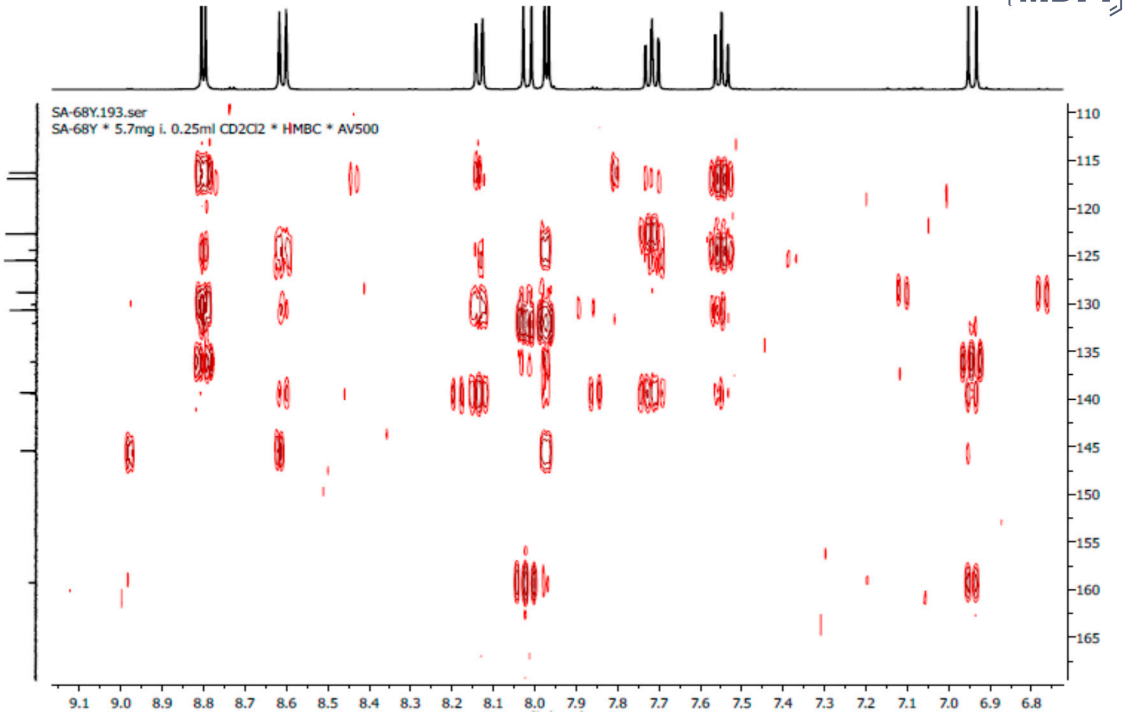

Figure S41. The ^{1}H-^{13}C HMBC NMR spectrum of canthin-6-one (**7**) observed at 500 and 125 MHz for CD_2Cl_2 solution at 25 °C. Assignment is given in Table S7.

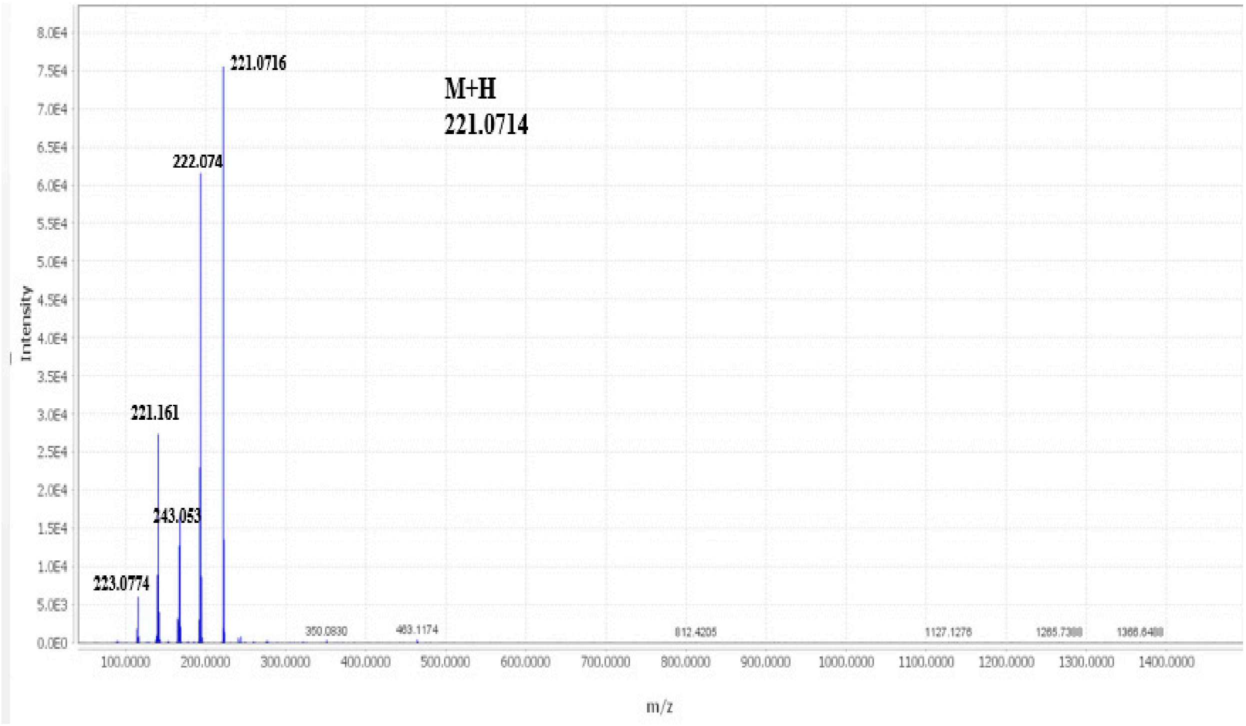

Figure S42. The ESIMS spectrum of canthin- 6- one (**7**).

1.3.8. 8-Oxochelerythrine (8)

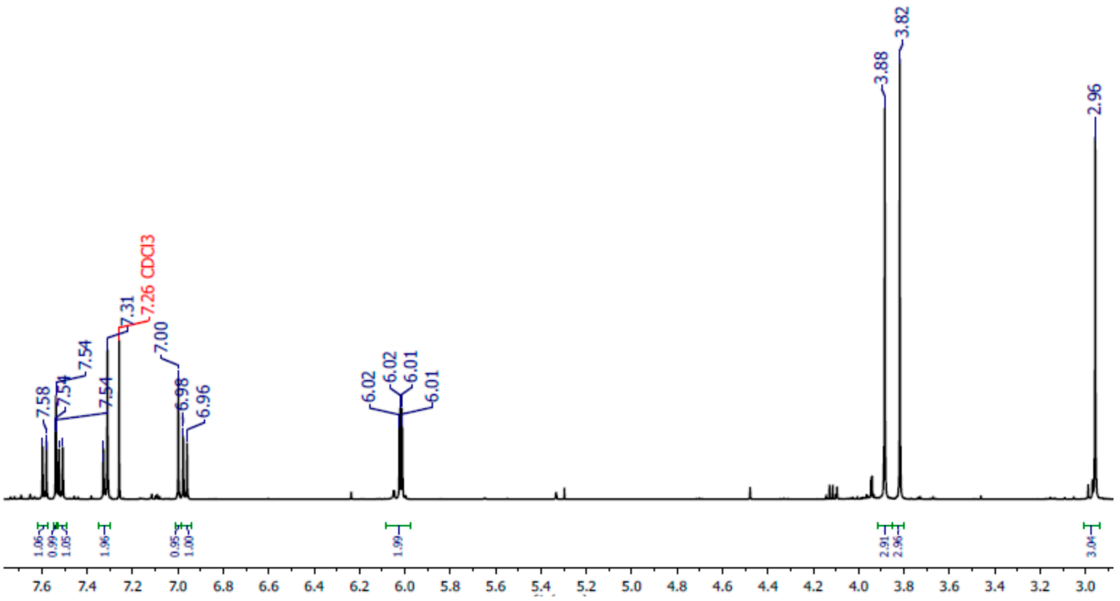

Figure 43. The ^{1}H NMR spectrum of 8-oxochelerythrine (**8**) observed at 500 MHz for $CDCl_3$ solution at 25 °C. Assignment is given in Table S8.

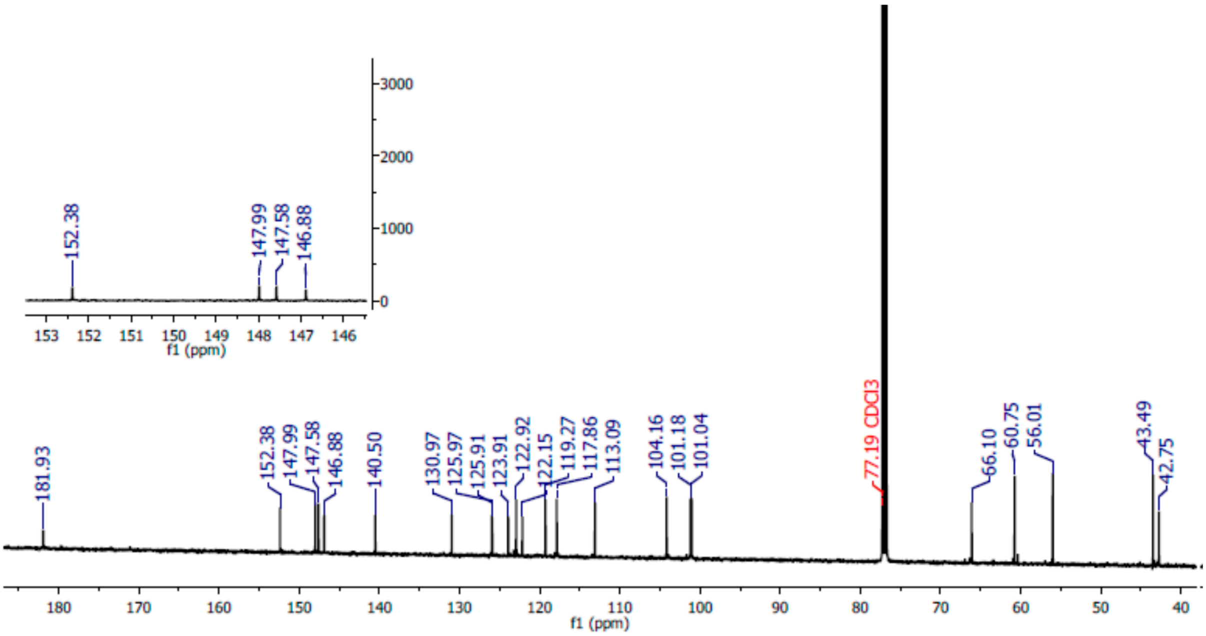

Figure S44. The ^{13}C NMR spectrum of 8-oxochelerythrine (**8**) observed at 125 MHz for $CDCl_3$ solution at 25 °C. Assignment is given in Table S8.

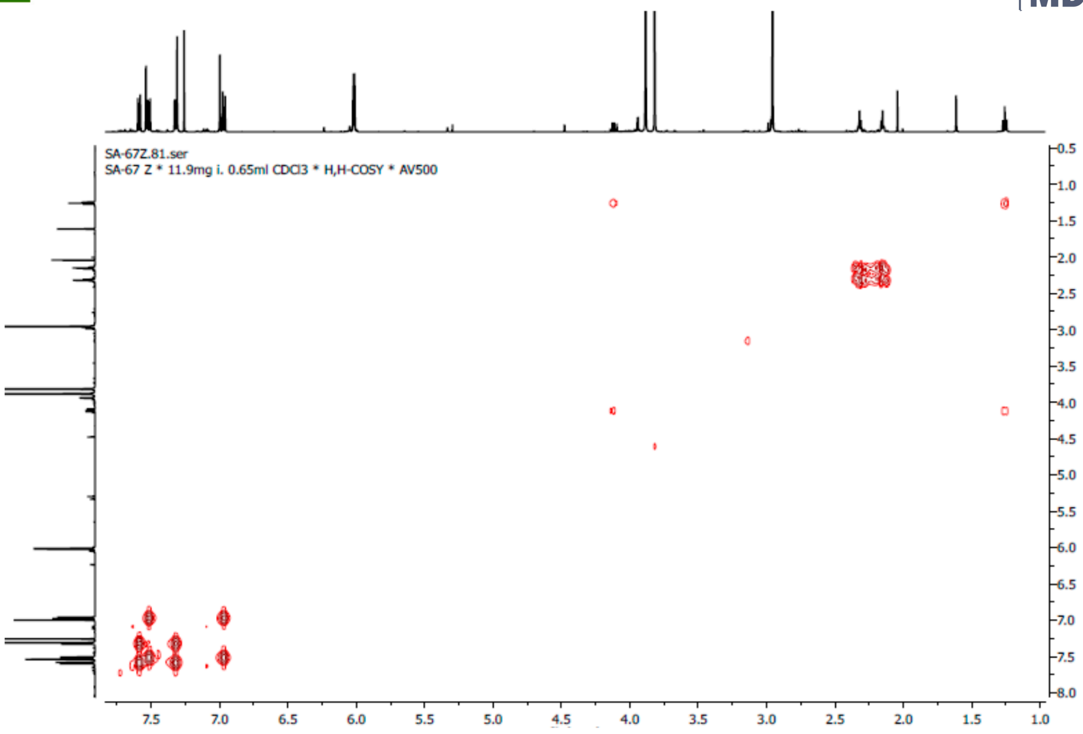

Figure S45. The ^{1}H-^{1}H COSY spectrum of 8-oxochelerythrine (**8**) observed at 500 MHz for $CDCl_3$ solution at 25 °C.

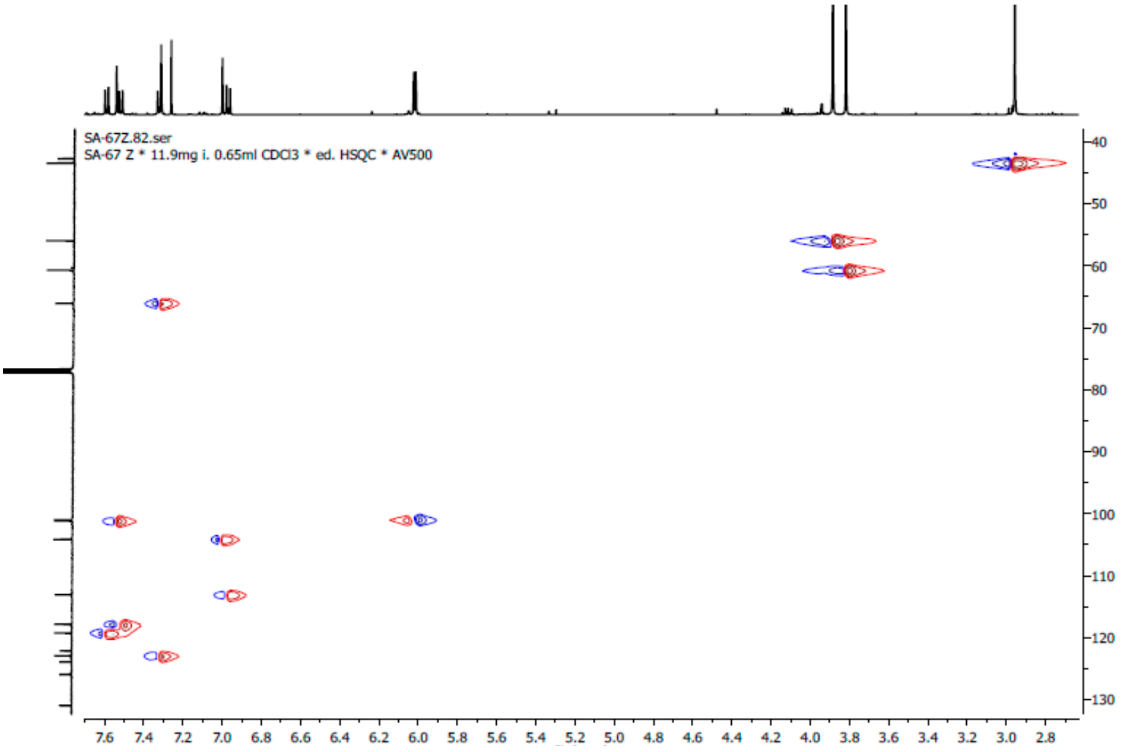

Figure S46. The ^{1}H-^{13}C HSQC NMR spectrum of 8-oxochelerythrine (**8**) observed at 500 and 125 MHz for $CDCl_3$ solution at 25 °C.

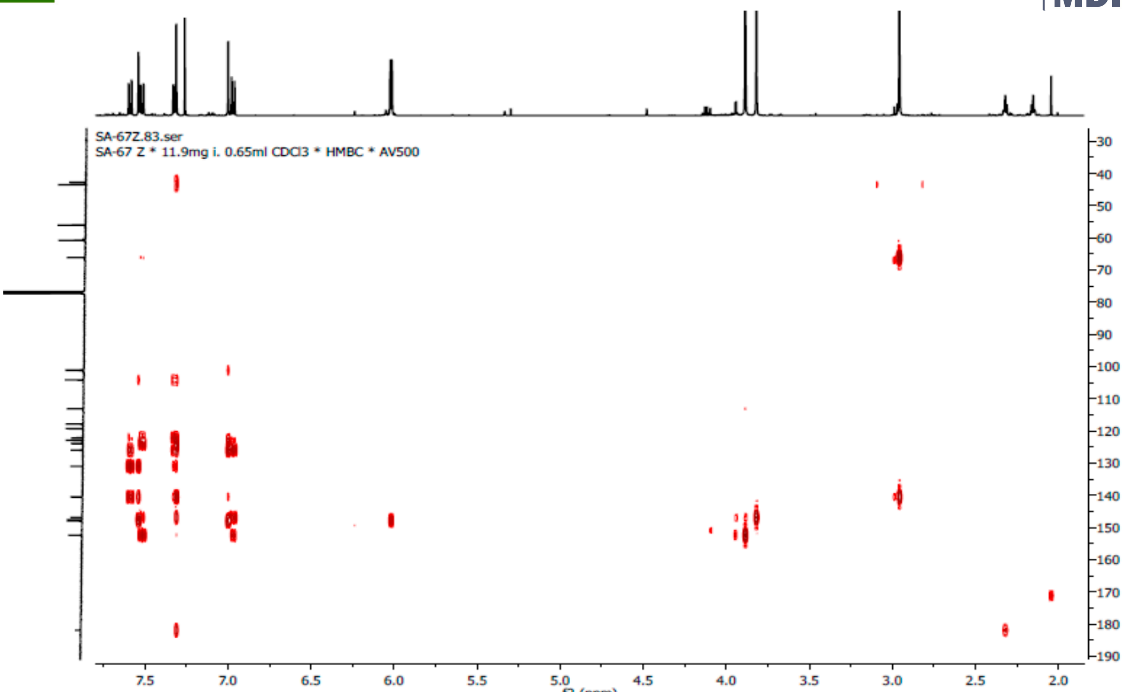

Figure S47. The ^{1}H-^{13}C HMBC NMR spectrum of 8-oxochelerythrine (**8**) observed at 500 and 125 MHz for $CDCl_3$ solution at 25 °C. Assignment is given in Table S8.

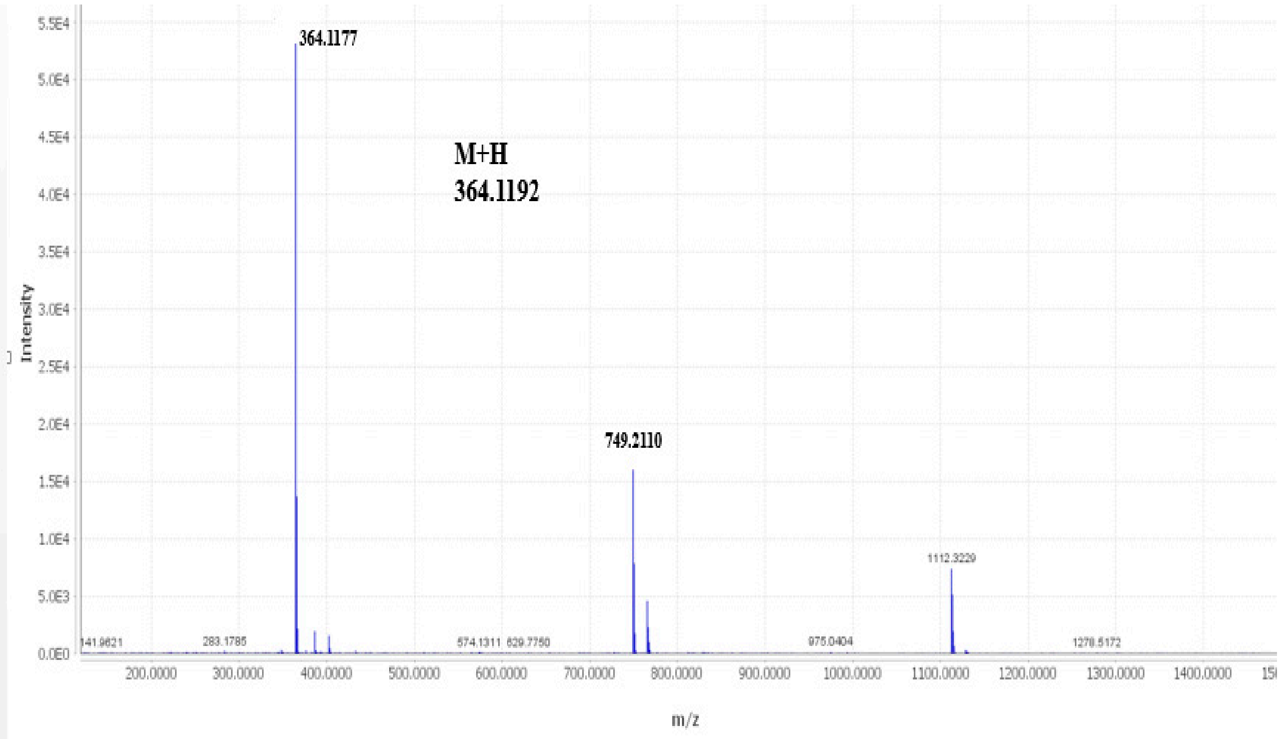

Figure S48. The ESIMS spectrum for 8-oxocheerythrine (**8**).

References

1. Yaouba S. Phytochemical investigation of selected plants in the families Anacardiaceae and Asteraceae for bioactive principles. University of Nairobi; 2018.
2. Ali H, Dixit S, Ali D, Alqahtani SM, Alkahtani S, Alarifi S. Isolation and evaluation of anticancer efficacy of stigmasterol in a mouse model of DMBA-induced skin carcinoma. Drug Des Devel Ther. 2015;9:2793.
3. Omosa LK, Mbogo GM, Korir E, Omole R, Seo E-J, Yenesew A, et al. Cytotoxicity of fagaramide derivative and canthin-6-one from *Zanthoxylum* (Rutaceae) species against multidrug resistant leukemia cells. Nat Prod Res. 2019;1–8.
4. Sreelekha M, Anto NP, Anto RJ, Shafi PM. Cytotoxicity of 6-acetonyldihydro-chelerythrin, arnottianamide and 6-(2-hydoxypropyl)-dihydrochelerythrine towards human cancer cell lines. 2014;
5. Adesina SK, Reisch J. Arnottianamide and other constituents of *Zanthoxylum gillettii* root. J Nat Prod. 1988;51(3):601–2.
6. Li N, Dai J-K, Liu D, Wang J-Y, Wang J-R. Synthesis and *in vitro* antibacterial activity of quaternization 10-methoxycanthin-6-one derivatives. 2019;
7. Buyinza D. Phytochemical investigation of *Zanthoxylum holstzianum* for antimicrobial. University of Nairobi; 2012.
